# Identification of Three Novel *Plasmodium* Factors Involved in Ookinete to Oocyst Developmental Transition

**DOI:** 10.3389/fcimb.2021.634273

**Published:** 2021-03-15

**Authors:** Chiamaka V. Ukegbu, George K. Christophides, Dina Vlachou

**Affiliations:** ^1^ Department of Life Sciences, Imperial College London, London, United Kingdom; ^2^ The Cyprus Institute, Nicosia, Cyprus

**Keywords:** ookinete development, malaria transmission, ookinete to oocyst transition, mosquito midgut invasion, *Plasmodium* sporogonic development, vector-parasite interactions

## Abstract

*Plasmodium falciparum* malaria remains a major cause of global morbidity and mortality, mainly in sub-Saharan Africa. The numbers of new malaria cases and deaths have been stable in the last years despite intense efforts for disease elimination, highlighting the need for new approaches to stop disease transmission. Further understanding of the parasite transmission biology could provide a framework for the development of such approaches. We phenotypically and functionally characterized three novel genes, *PIMMS01*, *PIMMS57*, and *PIMMS22*, using targeted disruption of their orthologs in the rodent parasite *Plasmodium berghei*. *PIMMS01* and *PIMMS57* are specifically and highly expressed in ookinetes, while *PIMMS22* transcription starts already in gametocytes and peaks in sporozoites. All three genes show strong phenotypes associated with the ookinete to oocyst transition, as their disruption leads to very low numbers of oocysts and complete abolishment of transmission. *PIMMS22* has a secondary essential function in the oocyst. Our results enrich the molecular understanding of the parasite-vector interactions and identify *PIMMS01*, *PIMMS57*, and *PIMMS22* as new targets of transmission blocking interventions.

## Introduction

Malaria remains a global public health problem with 228 million cases in 2018, causing 405,000 deaths (WHO World Malaria Report 2019). This deadly disease is caused by parasites of the genus *Plasmodium* transmitted by the bite of infected female *Anopheles* mosquitoes. *P. falciparum* is the most virulent of the human malaria parasites, responsible for most of malaria-associated deaths especially of children under 5 years old and first-time pregnant women. Transmission begins upon ingestion by a female *Anopheles* mosquito of a blood meal containing male and female gametocytes from an infected human. In the mosquito midgut, the gametocytes differentiate into male and female gametes that then mate to form the zygote. Within 24 h, the zygote, through several stages of development, differentiates into a crescent shaped motile ookinete that escapes the blood bolus and its encasing peritrophic matrix and invades and traverses the midgut epithelium. On the basal side of the midgut, the ookinete transforms into a sessile, replicative oocyst where thousands of sporozoites are produced. Oocysts burst and sporozoites released into the haemocoel reach the salivary glands from where they are transmitted to a new host during a subsequent mosquito bite.

The ookinete-to‐oocyst developmental transition in the mosquito midgut is the most critical stage of the entire parasite transmission cycle. The ingested parasite populations suffer major losses during this stage resulting in very few oocysts and, in most cases, termination of transmission. This stage is therefore a good target of interventions aiming to control disease transmission (Smith et al., 2014). Genome scale transcriptomic studies have shed important insights into *Plasmodium* gene expression and regulation that accompanies developmental stage transitions and developmental processes of the parasite (Le Roch et al., 2003; Hall et al., 2005; Otto et al., 2010; Otto et al., 2014; Lasonder et al., 2016; Yeoh et al., 2017; Reid et al., 2018). We have previously used DNA microarrays to identify transcriptional profiles driving the under characterized gametocyte-ookinete-to-oocyst developmental transitions in the rodent model parasite *Plasmodium berghei* (Akinosoglou et al., 2015); and more recently single cell RNA sequencing in *P. berghei* has also revealed genes differentially expressed during ookinete-to-oocyst developmental transition (Howick et al., 2019).

While rodent models are routinely employed in study parasite development in the mosquito vector, due to their genetic accessibility and tractability, functional differences in the utilization of genes between rodent and human parasites may be important especially with regards to specific interactions with the vector (Dong et al., 2006; Simões et al., 2017). Indeed, to cope with the live and changing host environment, *Plasmodium* has evolved the ability to undertake transcriptional variation for its survival and transmission (Rovira-Graells et al., 2012; Waters, 2016). *P. falciparu*m utilizes and infects about 60–70 *Anopheles* species, therefore, there would be a necessity to adapt to the different environments of each of these mosquito species, in particular with regards to differences in mosquito immune responses, behavior, and physiology (White et al., 2011; Mitri et al., 2015; Costa et al., 2018; Lefevre et al., 2018). For these reasons, we have developed an operational framework where *P. falciparum* genes potentially involved in parasite interactions with the vector are identified using genome-wide transcriptomic studies in near field settings and then prioritized for genetic and functional characterization in laboratory mosquito infections with *P. berghei*.

Here, we selected from our dataset three *P. falciparum* genes that exhibit highly abundant transcripts 24 h post blood feeding (hpbf) in the midguts of *Anopheles coluzzii* mosquitoes. These genes have been part of a large reverse genetic *P. berghei* screen to identify genes that function during parasite infection of the mosquito midgut, designated as *Plasmodium* Infection of the Mosquito Midgut Screen (PIMMS). We have previously reported the characterization of *PIMMS2* that encodes an ookinete membrane-associated subtilisin-like protein involved in midgut traversal (Ukegbu et al., 2017) and *PIMMS43* that encodes another ookinete membrane protein aiding in resistance to responses of the mosquito complement-like system (Ukegbu et al., 2020). Now we report the identification and characterization of three additional genes, *PIMMS01*, *PIMMS57*, and *PIMMS22*, which are highly transcribed and translated in the ookinete; with *PIMMS22* also highly expressed at both the gametocyte and sporozoite stages. PIMMS01 and PIMMS57 are putatively secreted and membrane-bound proteins, respectively, while PIMMS22 does not include any known secretory or membrane association signals but localizes on the periphery of ookinetes and sporozoites, possibly the inner membrane complex. An earlier study by Zheng and colleagues designated *PIMMS57* as *PSOP26* (Zheng et al., 2016) and showed the antibodies against *P. berghei* PIMMS57 can affect ookinete maturation and malaria transmission. We demonstrate that none of these genes has a role in ookinete development but are all important for the ookinete-to-oocyst developmental transition in the midgut and disease transmission.

## Materials and Methods

### Ethics Statement

Animal procedures were carried out in accordance with the Animal (Scientifics Procedures) Act 1986 under the UK Home Office Licenses PPL70/8788.

### Sequence Analysis

Plasmodium protein sequences were retrieved from PlasmoDB (http://plasmodb.org/plasmo/) and apicomplexan parasite sequences were retrieved from UniProt. Alignment was carried out using Clustal Omega and visualized in the BioEdit sequence alignment editor program. Signal peptide and transmembrane domains were predicted using SignalP and Phobius (Käll et al., 2007; Armenteros et al., 2019).

### *P. falciparum* Culturing and Mosquito Infections

*P. falciparum* NF54 was cultured as described previously (Habtewold et al., 2019). Briefly, ABS and gametocytes were cultured using human RBCs of various blood groups in the following order of preference: O+ male, O+ female, A+ male, and A+ female. Asexual cultures were set up in 10 ml complete medium [RPMI-1640, 0.05 g/L Hypoxanthine, 0.3 mg/L L-glutamine, 10% (v/v) sterile human serum of A+ serotype] containing a final volume of 500 μl of hRBCs (0.3%–4% infection). Cultures were gassed with “malaria gas” (3% O_2_/5% CO_2_/92% N_2_) and incubated at 37°C. Gametocyte cultures were set up by dilution of a 3-4% ring stage asexual culture to 1% ring forms in a final volume of 8 ml complete medium and supply of fresh hRBCs. The cultures underwent daily exchange of around 75% of the media, gassed with malaria gas and incubated at 37°C until day 14. Mosquitos were infected with *P. falciparum* by SMFAs as described previously (Habtewold et al., 2019). Briefly, Giemsa staining was used to assess gametocyte density and *in vitro* exflagellation was used to assess viability of stage V male gametocytes. In a pre-warmed tube, the gametocyte cultures were pooled in a final volume of 300 μl containing 20% (v/v) uninfected serum-free hRBCs and 50% (v/v) heat-inactivated human serum. This mixture was then transferred to pre-warmed mosquito feeders kept a constant temperature of 37°C.

### *P. berghei* Strains and Cultivation

*P. berghei* lines used were the reference parent line of *P. berghei* ANKA 2.34 (*cl15cy1*) and the *507m6cl1* (*c507*) line that contains *GFP* integrated into the *230p* gene locus (*PBANKA_0306000*) without a drug selectable marker and constitutively expresses *GFP* under the control of the EF1 alpha promoter (Janse et al., 2006a). All parasite lines were maintained in 8-10-week-old CD1 and/or TO female mice by serial passaging. *P. berghei* mixed blood stages, gametocytes, and ookinetes were cultured and purified as described previously (Janse and Waters, 1995; Beetsma et al., 1998).

### Quantitative RT-PCR

Total RNA was extracted from *P. berghei* and *P. falciparum* parasites or *A. coluzzii* infected with *P. berghei* or *P. falciparum* using Trizol reagent (ThermoFisher) according to the manufacturer’s instructions. cDNA was synthesized using the PrimeScript Reverse Transcription Kit (Takara) after Turbo DNase (ThermoFisher) treatment. For qRT-PCR, SYBR green (Takara) and gene specific qRT-PCR primers (**Table S6**) were used according to the manufacturer’s guidelines. Gene expression was normalized against *GFP* in *P. berghei* and against *P. falciparum* arginyl-tRNA synthetase in *P. falciparum* using the ΔΔCt method.

### Antibody Production

Rabbit polyclonal antibodies against peptides of the deduced proteins PIMMS01 and PIMMS57 were raised and purified from the serum of an immunized rabbit (Eurogentec): α-Pfc01 targets the PfPIMMS01 peptide EKHKDSTKWDKSYSF (aa 72–86); α-Pbc57 targets the PbPIMMS57 the N-terminal peptide SNDSNYEDRDNAPNR (aa 48–62); and α-Pfc57, targets the N-terminal peptide EQRVRDEGRENNRRS (aa 111–125.)

### Western Blot Analysis

Soluble cell lysates were prepared by suspending purified parasite pellets in cell lysis buffer (5 mM Tris, 150 mM NaCl) containing protease inhibitors. Triton X-100 soluble cell lysates were prepared by suspending purified parasite pellets in Triton X-100 cell lysis buffer (5 mM Tris, 150 mM NaCl, 1% v/v Triton X-100) containing protease inhibitors. Triton X-100 insoluble cell fractions/whole cell lysates were prepared by suspending parasite pellets or infected midguts in reducing (3% v/v 2-mercapthoethanol) Laemmli buffer. Protein samples were then boiled under reducing (3% v/v 2-mercapthoethanol) conditions in Laemmli buffer and separated using sodium dodecyl sulfate polyacrylamide gel electrophoresis. Separated proteins were then transferred to a PVDF membrane (GE Healthcare). Proteins were detected using Goat α-GFP (Rockland chemicals) (1:100), mouse α-tubulin (Sigma), and 3D11 mouse monoclonal α-PbCSP (Potocnjak et al., 1980) (1:1000), α-Pbc57 (1:100), α-Pfc01 (1:50), α-Pfc57 (1:50), and α-Pfs25 (Barr et al., 1991), (1:100) antibodies. Secondary horseradish peroxidase (HRP) conjugated IgG, goat α-mouse IgG antibodies (Promega), and donkey α-goat IgG (Abcam) were used at 1: 10,000 and 1: 5,000 dilutions, respectively. All primary and secondary antibodies were diluted in 3% milk-PBS-Tween (0.05% v/v) blocking buffer.

### Indirect Immunofluorescence Assay

For IFAs on blood bolus parasites, the blood bolus was collected from dissected midguts of mosquitoes at 2 and 19 hpbf. The blood bolus was washed in PBS and fixed in 4% paraformaldehyde (PFA) for 10 min. The fixed parasites were smeared on a glass slide, permeabilized with 0.2% (v/v) Triton X-100, and blocked with 3% (w/v) bovine serum albumin Purified gametocytes and *in vitro* ookinetes were fixed, permeabilized and blocked as above. For IFAs on midgut sporozoites at 15 dpbf, infected midguts were dissected, and tissues were homogenized to release sporozoites. Sporozoites were fixed, blocked and permeabilized as that above. For IFAs on ookinetes invading the midgut epithelium, the midguts of mosquitoes at 26 hpbf were dissected, and the blood boluses were discarded. The midgut epithelium was fixed in 4% PFA in PBS for 45 min and washed thrice in PBS for 10 min each. Midgut epithelium was permeabilized and blocked for 1 h in 1% w/v BSA, 0.1% v/v Triton X-100 in PBS blocking solution. Samples were then stained in blocking solution with primary antibodies (α-GFP, 1:100; 13.1 mouse monoclonal α-P28 (Winger et al., 1988), 1:1000; and α-PbCSP 1:100; α-Pfc01, 1:100; α-Pfc57, 1:100; 4B7 mouse monoclonal α-Pfs25, 1:100. Alexa Fluor (488 and 568) conjugated secondary antibodies specific to goat or mouse (ThermoFisher) were used at a dilution of 1:1000. 4′,6-diamidino-2-phenylindole (DAPI) was used to stain nuclear DNA. Images were acquired using a Leica SP5 MP confocal laser-scanning microscope. Images underwent processing by deconvolution using Huygens software and were visualized using Image J.

### Generation of Transgenic Parasites

For *GFP* tagging of *Pbc01* in the *2.34* line, a 603 bp ApaI/HindIII 5’ and a 770 bp EcoRI/BamHI 3’ homology arm region were amplified from *P. berghei 2.34* genomic DNA using the primer pairs P1/P2 and P3/P4, respectively. For *GFP* tagging of *Pbc57* in the *2.34* line, a 919 bp ApaI/SacII 5’ homology arm corresponding to the most 3’ region of the CDS without the stop codon and a 359 bp XhoI/XmaI 3’ homology arm region corresponding to the *3’ UTR* of the gene were amplified using the primer pairs P5/P6 and P7/P8, respectively. For *GFP* tagging of *Pbc22* in the *2.34* line, a 753 bp ApaI/HindIII 5’ homology arm corresponding to the most 3’ region of the CDS without the stop codon and a 530 bp EcoRI/BamHI 3’ homology arm region corresponding to the *3’ UTR* of the gene were amplified using the primer pairs P9/P10 and P11/P12, respectively. The *Pbc01* and *Pbc22* fragments were cloned into the pBS-TgDHFR vector which carries a modified *Toxoplasma gondii* dihydrofolate gene (TgDHFR/TS) cassette that confers resistance to pyrimethamine (Dessens et al., 1999). The *Pbc57* fragments were cloned into plasmid pL0035 which carries the *hDHFR* selection cassette (Braks et al., 2006). Finally, to put *GFP* tag in frame with the 3’ region of the CDS, a HindIII or SacII *GFP-P. berghei DHFR 3’UTR* fragment was amplified from the pL00018 vector (MRA-787, MR4) using primers P13/P14 or P15/P16, respectively.

For *3XHA* tagging of *Pbc01* in the c*507* line, a 687 bp ApaI/SacII 5’ homology arm corresponding to the some of the *5’UTR*, the whole CDS without the stop codon and the 3XHA tag and a 2051 bp XhoI/XmaI 3’ homology arm region corresponding to the *3’ UTR* of the gene was amplified from genomic DNA using the Gibson assembly primer pairs P43/P44 and P45/P46, respectively. For *3XHA* tagging of *Pbc57* in the c*507* line, a 1003 bp ApaI/SacII 5’ homology arm corresponding to the most 3’ region of the CDS without the stop codon and the 3XHA tag and a 359 bp XhoI/XmaI 3’ homology arm region corresponding to the *3’ UTR* of the gene was amplified using the Gibson assembly primer pairs P47/P48 and P49/P50 respectively. The *Pbc01* and *Pbc57* fragments were cloned into plasmid pL0035 *via* Gibson assembly.

For partial disruption of *Pbc01* in the *c507* line, a 432 bp ApaI/HindIII 5’ homology arm and a 1102 bp EcoRI/BamHI 3’ homology arm was amplified using the primer pairs P24/P25 and P26/P27, respectively. For partial disruption of *Pbc57* in the *c507* line, a 590 bp ApaI/HindIII 5’ homology arm and a 684 bp EcoRI/BamHI 3’ homology arm was amplified using the primer pairs P28/P29 and P30/P31, respectively. For partial disruption of *Pbc22* in the *c507* line, a 560 bp ApaI/HindIII 5’ homology arm and a 712 bp EcoRI/BamHI 3’ homology arm was amplified using the primer pairs P32/P33 and P34/P35, respectively. These fragments were cloned into the pBS-TgDHFR vector. The targeting cassettes by ApaI/BamHI digestion allows knockout of 39% of *Pbc01* CDS, 70% of *Pbc57* CDS, and 80% of *Pbc22* CDS.

Transfection of linearized constructs, selection of transgenic parasites and clonal selection was carried out as described previously (Janse et al., 2006b).

### Genotypic Analysis of Transgenic Parasites

Blood stage parasites were purified by removal of white blood cells using hand packed cellulose (Sigma) columns. Parasites were released using red blood cell lysis buffer (0.17 M NH_4_Cl) on ice for 20 min. Genomic DNA was extracted from parasites using the DNeasy kit (Qiagen). Integration events or maintenance of the unmodified locus was detected by PCR on genomic DNA using primers listed in **Table S6**. For PFGE, blood stage parasites within agarose plugs were lysed in lysis buffer (1X TNE, 0.1 M EDTA pH 8.0, 2% (v/v) Sarkosyl, 400 μg/ml proteinase K) to release nuclear chromosomes. PFGE separated chromosomes (Run settings: 98 volts, 1–5 mins pulse time for 60 h at 14°C) were then subjected to Southern blot analysis using a probe targeting the *TgDHFR/TS-P. berghei DHFR 3’UTR*, obtained by HindIII and EcoRV digestion of the pBS-TgDHFR plasmid.

### Phenotypic Assays

Exflagellation assays were performed as described previously (Akinosoglou et al., 2015). Briefly, blood from a high gametocytemic mouse was added in a 1:40 ratio to ookinete medium (RPMI 1640, 20% v/v FBS, 100 μM xanthurenic acid, pH 7.4), and exflagellation was counted in a standard hemocytometer under a light microscope. Conversion assays were performed as previously described (Akinosoglou et al., 2015). Briefly, *in vitro* cultivated ookinetes were resuspended in 50 μl of fresh ookinete and incubated with a Cy3-labeled 13.1 mouse monoclonal α-P28 (1:50 dilution) for 20 min on ice. The conversion rate was calculated as the percentage of Cy3 positive ookinetes to Cy3 positive macrogametes and ookinetes.

Ookinete motility assays were performed as described previously (Moon et al., 2009). Briefly, on a glass slide, 24 h *in vitro* ookinete culture was incubated with Matrigel (BD biosciences), and allowed to set at RT for 30 min. On a Leica DMR fluorescence microscope and a Zeiss Axiocam HRc camera controlled by the AxioVision (Zeiss) software, time-lapse images (1 frame every 5 s for 10 min) of ookinetes were obtained. Using the manual tracking plugin in the Icy software (http://icy.bioimageanalysis.org/), the speed of individual ookinetes was measured.

*A. coluzzii* mosquitoes were fed by direct blood feeding as previously described (Sinden, 1997) on mice with parasitemia of 4%–5% and gametocytemia of 1%–2%. Midguts tissues were dissected at 7–10 dpbf and fixed in 4% PFA in PBS and mounted in Vectashield^®^ (VectorLabs). Oocysts or melanized ookinetes were counted using light and/or fluorescence microscopy. 25-30 P*. berghei* infected *A. coluzzii* midguts or salivary glands at 15 and 21 dpbf respectively were homogenized and oocyst and salivary gland sporozoites counted using a standard hemocytometer. Finally, in mosquito to mouse transmission assays, at least 30 P*. bergh*ei infected mosquitoes at 21 dpbf were allowed to feed on two to three anaesthetized C57/BL6 mice for 15 min. Parasitemia was monitored until 14 days post mosquito bite by Giemsa staining of blood smears.

### Ookinete Injections in Mosquito Haemocoel

Ookinete injections were carried out as described previously (Bushell et al., 2009). Briefly, the concentration of ookinetes from 24 h *in vitro* cultures was adjusted with RPMI 1640, and this was injected using glass capillary needles and the Nanoject II microinjector (Drummond Scientific) into the thorax of *A. coluzzii* mosquitoes at a final concentration of 800 ookinetes per mosquito.

### Gene Silencing

Total RNA extracted from *A. coluzzii* midgut infected with *P. berghei c507* at 24 hpbf was used to prepare cDNA. The cDNA was used in conjunction with primers reported in (Habtewold et al., 2008) to amplify CTL4. DsRNA was then produced using the resulting PCR product and the T7 high yield transcription kit (ThermoFisher). 0.2 μg of purified dsRNA in 69 nl was injected into the thorax of *A. coluzzii* mosquitoes using glass capillary needles and the Nanoject II microinjector. Two to three days post injected mosquitoes were then infected with *P. berghei*.

### Statistical Analysis

Statistical analyses were performed using GraphPad Prism v8.0. Statistical analyses for exflagellation, ookinete conversion and motility assays were performed using a two-tailed, unpaired Student’s t-test. For statistical analyses of the oocyst or melanized parasite load, P-values were calculated using the Mann-Whitney test.

## Results

### *In Silico* Analysis

Based on their expression profiles in a *P. falciparum* transcriptomics dataset and mutant phenotypes in a *P. berghei* high throughput reverse genetics screen, three parasite genes were selected for further characterization, including targeted disruption and detail phenotypic analysis in *P. berghei*. These genes were *PF3D7_0112100* in *P. falciparum* and *PBANKA_*0201700 in *P. berghei*, *PF3D7_1244500* in *P. falciparum* and *PBANKA_1457700* in *P. berghei*, and *PF3D7_0814600* in *P. falciparum* and *PBANKA_1422900* in *P. berghei*; designated as *PIMMS01*, *PIMMS57*, and *PIMMS22*, respectively.

*PfPIMMS01* encodes a 163 amino acid-long protein (19 kDa) with a predicted signal peptide (aa 1–27; probability according to SignalP and Phobius 0.688 and 0.904, respectively). Its *P. berghei* orthologue, *PbPIMMS01*, encodes a much shorter 85 amino acid-long protein (9 kDa) and also contains a predicted signal peptide (aa 1–26; probability according to SignalP and Phobius 0.826 and 0.978, respectively). This suggests that both proteins are putatively secreted. While the central part of the deduced protein is highly conserved between all PIMMS01 orthologues, all rodent PIMMS01 proteins are shorter than their orthologues in human parasites, lacking the entire second half of the protein (**Figure S1**). InterPro domain analysis revealed no recognizable domain, and BLAST searches showed that the protein is *Plasmodium* specific.

*PfPIMMS57* encodes a 810 amino acid-long (94 kDa) protein with a predicted signal peptide (aa 1–26; probability according to SignalP and Phobius 0.697 and 0.657, respectively) that overlaps with a putative transmembrane domain albeit with low probability according to Phobius (0.343). A second transmembrane domain close to the carboxy terminus of PfPIMMS57 (aa 677–697) is predicted with very high probability (0.874). The *P. berghei* orthologue, *PbPIMMS57*, encodes a protein of 774 amino acid-long (90 kDa) protein with two putative transmembrane domains (aa 6–23 and aa 633–653) predicted with high probability (0.984 and 0.967, respectively). These data suggest that both proteins are putatively membrane-bound. Higher sequence conservation between PIMMS57 orthologues is observed in the second half of the proteins compared to the first half, pointing to a conserved functional role served by this region (**Figure S2**). A previous *in silico* analysis identified in PfPIMMS57, two *P. falciparum* serine/threonine protein phosphatase type I catalytic (PfPP1c) binding motifs, the RVxF motif, RRKVNF (aa 347–352) and the Fxx[RK]x[RK] motif, FNKILKR (aa 488–494) (Hollin et al., 2016). These motifs are not conserved in the other PIMMS57 orthologues.

Finally, *PfPIMMS22* encodes a 393 amino acid (45 kDa) protein and *PbPIMMS22* encodes a 393 aa (44 kDa) protein, both with no predicted signal peptide or transmembrane domain. The protein is highly conserved amongst *Plasmodium* orthologues with sequence identity ranging from 96% in PyPIMMS22 to 76% and 80% in PfPIMMS22 and PvPIMMS22, respectively (**Figure S3**). PyPIMMS22 (PY17X_1424900) was previously identified in salivary gland sporozoites through a subtractive hybridization (SSH) profiling and termed sporozoite protein S15 (Kaiser et al., 2004). The same protein was also identified in midgut oocyst sporozoites as an interacting partner to the apicomplexan specific RNA-binding protein, ALBA4, which is involved in mRNA regulation in gametocyte and midgut oocyst sporozoite development (Muñoz et al., 2017). PIMMS22 homologues are found in other apicomplexan parasites including *Toxoplasma gondii*, *Neospora caninum* and *Eimeria* with sequence identities to PIMMS22 ranging from 36% to 42% (**Figure S4**). InterPro domain analysis revealed no recognizable domain in PIMMS22.

### Transcription Profiles

**Figure 1:**
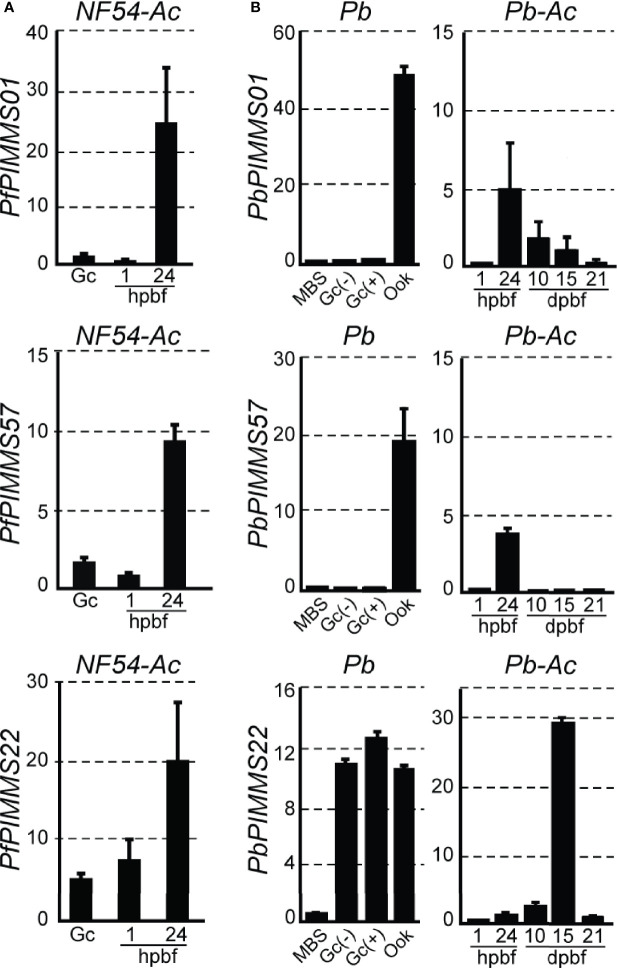
*PIMMS01*, *PIMMS57*, and *PIMMS22* transcriptional profiles. **(A)** Relative abundance of *PfPIMMS01*, *PfPIMMS57*, and *PfPIMMS22* transcripts in purified *P. falciparum in vitro* cultured gametocytes, and *A. coluzzii* mosquito infected midguts at 1 and 24 hpbf, as determined by qRT-PCR in *NF54* parasite populations and normalized against the Arginyl-tRNA synthetase. **(B)** Relative abundance of *P. berghei PIMMS01*, *PbPIMMS57*, and *PbPIMMS22* transcripts in purified blood stages and *in vitro* produced ookinetes, and in *A*. *coluzzii* mosquito stages, as determined by qRT-PCR in the *c507* line and normalized against the constitutive expressed *GFP*. In all panels. each bar is the average of three biological replicates. Error bars indicate SEM. MBS, mixed blood stages; Gc, gametocytes; Gc(+), activated gametocytes; Gc(-), non-activated gametocytes; Ook, ookinetes; hpbf, hours post blood feeding; dpbf, days post blood feeding.

We searched a DNA microarray dataset of *A. coluzzii* midguts infected with *P. falciparum* field isolates from Burkina Faso to examine the transcriptional profiles of the three genes under study. *PfPIMMS01* and, to a lesser extent, *PfPIMMS57* were lowly transcribed 1 h post blood-feeding (hpbf) and their transcription peaked 24 hpbf, while *PfPIMMS22* expression started in gametocytes and continued at high levels 24 hpbf. We corroborated these data with quantitative real-time RT-PCR (qRT-PCR) in laboratory *P. falciparum* NF54 cultured gametocytes and in NF54 infections of *A. coluzzii* 1 and 24 hpbf (**Figure 1A**). Very low levels of *PfPIMMS01* and *PfPIMMS57* transcripts were detected in gametocytes and in *A. coluzzii* midguts 1 hpbf, peaking at 24 hpbf.

Transcription of the *P. berghei* orthologous genes was also analyzed using qRT-PCR (**Figure 1B**). In this assay, the *P. berghei* line *ANKA507m6cl1* that constitutively expresses GFP was used (Janse et al., 2006a); hereafter referred to as *c507*. *PbPIMMS01* and *PbPIMMS57* transcripts were highly abundant in purified mature ookinetes (Ook) and not detectable in mixed blood stages (MBS) and purified gametocytes. While *PbPIMMS57* appears to be specific for ookinetes, low levels of *PbPIMMS01* transcripts are also detected in mature oocysts and midgut sporozoites at 10 days and 15 days post blood-feeding (dpbf). Like *PfPIMMS22*, expression of *PbPIMMS22* starts in gametocytes and continues at high levels 24 hpbf and peaks in midgut sporozoites 15 dpbf (**Figure 1B**). Low abundance *PbPIMMS22* transcripts detected in MBS is likely due to expression in gametocytes. These results generally agree with those in published RNA-sequencing data from blood stages and ookinetes (Otto et al., 2014; Yeoh et al., 2017).

### Protein Expression and Localization

Protein expression was assessed by endogenous *GFP* tagging of the three *P. berghei* genes *via* double crossover homologous recombination in the ANKA 2.34 line. The generated transgenic parasites were named *c01::gfp*, *c57::gfp*, and *c22::gfp* (**Figure S5**). *P. berghei PIMMS01* and *PIMMS57* genes were also terminally tagged with the 3xHA (hemagglutinin) tag in the *c507* line, and transgenic parasites were named *c01::3xha* and *c57::3xha* (**Figure S6**). We also generated rabbit polyclonal antibodies raised against peptides of the deduced proteins in both *P. berghei* and *P. falciparum*. Of all antibodies, those that produced data and are analyzed below were: α-Pfc01, PfPIMMS01 peptide aa 72-86; α-Pbc57, PbPIMMS57 peptide aa 48-62; and α-Pfc57, PfPIMMS57 peptide aa 111–125.

Mosquito stage development of transgenic GFP-tagged *P. berghei* lines was assessed by counting midgut sporozoite numbers 15 dpbf. Amongst the three transgenic parasites, only *c22::gfp* produced wild type (wt) number of midgut sporozoites (**Table S1**). Lines *c01::gfp* and *c57::gfp* yielded severely reduced sporozoite numbers, suggesting that insertion of GFP compromises the function of the two proteins. Similar to their GFP-tagged counterpart and concomitant gene knockout (see next section) parasite lines, both HA-tagged transgenic lines, showed severely reduced oocyst numbers compared to the *c507* reference parasite, as assessed by counting oocyst numbers 8 dpbf (**Table S2**). This phenotype suggests that insertion of the 3xHA tag compromises the function of the two proteins.

#### PIMMS01 Expression and Localization

**Figure 2:**
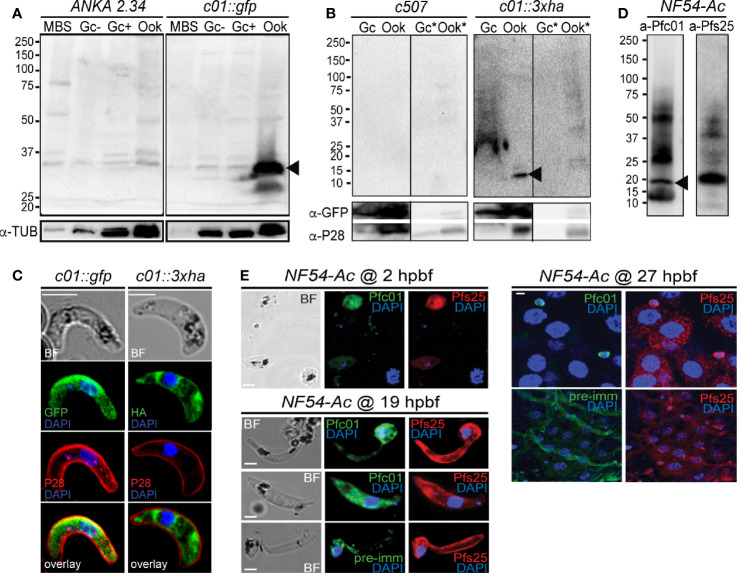
PIMMS01 protein expression and localization. **(A)** Western blot analysis using α-GFP antibody on whole cell lysates of *P. berghei c01::gfp* parasites. The PbPIMMS01::GFP fusion protein band is indicated with a black arrowhead. The ANKA *2.34* parental parasite line was used as a negative control. Tubulin was used as a loading control. MBS, mixed blood stages; Gc(-), non-activated gametocytes; Gc(+), activated gametocytes; Ook, ookinetes. **(B)** Western blot analysis using the α-HA antibody on Triton X-100 soluble lysates of *in vitro* gametocyte (Gc) and ookinetes (Ook) and Triton X-100 insoluble lysates (Gc*, Ook*) of *c01::3xha*. The c*507* reference line (left panel) was used as a negative control. PbPIMMS01::HA fusion protein band is indicated with a black arrowhead. GFP and P28 were used as a loading and a stage specific control, respectively. **(C)** Immunofluorescence assays on ookinetes of PIMMS01 tagged with GFP (left panel) and 3xHA (right panel) stained with α-GFP or α-HA (green), respectively, as well as with the ookinete surface α-P28 (red). DNA was stained with DAPI. Images are de-convoluted projections of confocal stacks. BF, bright field; Scale bars, 5 μm. **(D)** Western blot analysis using α-Pfc01 antibody on whole cell lysates of *coluzzii* ****midguts at 22 h post blood feeding (hpbf). The PfPIMMS01 protein band is indicated with a black arrowhead. Tubulin was used a loading control for Gc and Sch while Pfs25 was used for 22 hpbf. **(E)** Immunofluorescence assays of *P. falciparum* NF54 parasites in mosquito blood bolus at 2 hpbf, ookinetes in mosquito blood bolus at 19 hpbf and ookinetes traversing the mosquito midgut epithelium at 27 hpbf, stained with α-Pfc01 (green) and the female gamete/zygote/ookinete α-Pfs25 (red) antibodies. Pre-immune (pre-imm) serum was used as negative control. DNA was stained with DAPI. Pre-immune serum was used as a negative control. BF, bright field; hpbf, hours post blood feeding. Scale bars, 5 μm.

Western blot analysis using an α-GFP antibody on the non-functional *c01::gfp* line confirmed high levels of PbPIMMS01::GFP fusion protein at the expected molecular weight (**Figure 2A**). The protein was found to be highly expressed in mature ookinetes of the *c01::gfp* line and be absent from MBS or gametocytes or any stage of the *ANKA 2.34* background parasite. Similarly, western blot analysis on the *c01::3xha* transgenic line using an α-HA antibody confirmed expression of the PbPIMMS01::3xHA fusion protein in the ookinete at the expected molecular weights and absence from gametocytes (**Figure 2B**). The protein was only detected in the Triton X-100 soluble fraction suggesting that it is not membrane associated.

Despite the strong evidence that the PbPIMMS01 GFP- and HA-tagged proteins are non-functional, these are still likely to be localised correctly. IFAs on the developmentally compromised *c01::gfp* and *c01::3xha* transgenic lines revealed strong cytoplasmic localization of both PbPIMMS01::GFP and PbPIMMS01::3xHA (**Figure 2C**), which indicates that the protein may be stuck in a secretory pathway and suggests that the native polypeptide may be associated with the ookinete outer membrane especially during invasion. Peptide antibodies raised against *P. berghei* PIMMS01 did not work in western blots and IFAs.

In *P. falciparum*, the α-Pfc01 antibody detected a band of about 20 kDa (predicted molecular weight of PfPIMMS01 is 19 kDa) in *A. coluzzii* midguts infected 22 h earlier with *P. falciparum* NF54 (**Figure 2D**). This band was not detected in NF54 cultured schizonts or gametocytes (**Figure S7**). In indirect immunofluorescence assays (IFAs) using the α-Pfc01 antibody, PfPIMMS01 was detected in gametes/early zygotes found in the blood bolus 2 hpbf, ookinetes in the mosquito blood bolus 19 hpbf, and ookinetes crossing the mosquito midgut epithelium (**Figure 2E**). Its localization varied from cytoplasmic in early developmental stages, mostly stages III and IV of the ookinete development, to totally peripheral in mature, midgut-crossing ookinetes.

#### PIMMS57 Expression and Localization

**Figure 3:**
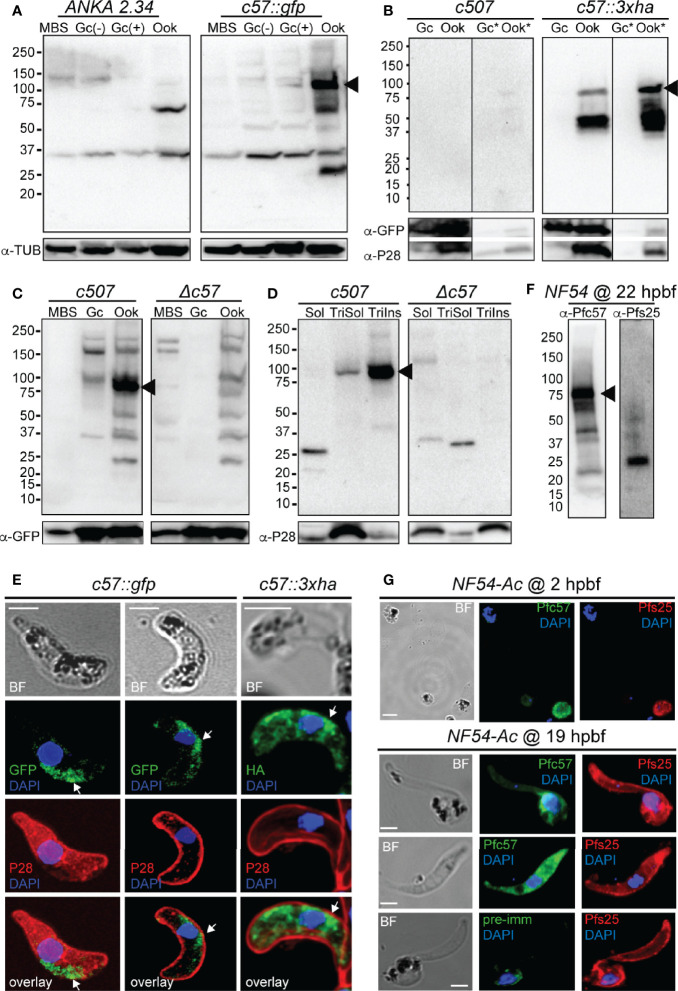
PIMMS57 protein expression and localization. **(A)** Western blot analysis using α-GFP antibody on whole cell lysates of the *P. berghei c57::gfp* parasites. The PbPIMMS57::GFP fusion protein band is indicated with a black arrowhead. The *ANKA 2.34* parental parasite was used as a negative control. Tubulin was used a loading control. MBS, mixed blood stages; Gc(-), non-activated gametocytes; Gc(+), activated gametocytes; Ook, ookinetes. **(B)** Western blot analysis using the α-HA antibody on Triton X-100 soluble lysates of *in vitro* gametocyte (Gc) and ookinetes (Ook) and Triton X-100 insoluble lysates (Gc*, Ook*) of *c57::3xha*. The c*507* reference line (left panel) was used as a negative control. PbPIMMS57::HA fusion protein band is indicated with a black arrowhead. GFP and P28 were used as a loading and a stage specific control, respectively. **(C)** Western blot analysis using the α-Pbc57 peptide antibody on whole cell lysates of MBS, Gc, and ookinetes of the *c507* parasite line. The PbPIMMS57 protein band is indicated with a black arrowhead. *Δc57* parasites were used as a negative control. GFP was used as a loading control. **(D)** Western blot analysis using the α-Pbc57 antibody on fractionated *in vitro* ookinetes. The PbPIMMS57 protein band is indicated with a black arrowhead. *Δc57* ookinetes were used as a negative control. P28 was used as a loading control. Soluble (Sol), Triton X-100 soluble (TriSol) and Triton X-100 Insoluble (TriInso) fractions are shown. **(E)** Immunofluorescence assays on ookinetes of PbPIMMS57 tagged with GFP and 3xHA stained with α-GFP or α-HA (green; white arrows) as well as with the ookinete surface α-P28 (red). DNA was stained with DAPI. Staining of the *c507* and *ANKA 2.34* parental parasites were used as negative controls for the HA and GFP staining, respectively. Images are de-convoluted projections of confocal stacks. BF, bright field; Scale bars, 5 μm. **(F)** Western blot analysis using α-Pfc57 antibody on whole cell lysates of *A*. *coluzzi* ****midguts at 22 hpbf. The PfPIMMS57 protein band is indicated with a black arrowhead. Pfs25 was used as a loading and stage specific control for 22 hpbf. **(G)** Immunofluorescence assays of *P. falciparum* NF54 parasites in mosquito blood bolus at 2 hpbf (top) and ookinetes in mosquito blood bolus at 19 hpbf (bottom), stained with α-Pfc57 (green) and the female gamete/zygote/ookinete α-Pfs25 (red) antibodies. Pre-immune serum was used as a negative control. DNA was stained with DAPI. Staining with pre-immune serum was used as a negative control. BF, bright field; Scale bars, 5 μm.

Using the non-functional *c57::gfp* parasite line and the α-GFP antibody in western blot analysis, high levels of the PbPIMMS57::GFP fusion protein were detected in mature ookinetes, at the expected molecular weight of 117 kDa (**Figure 3A**). This band was not detected in MBS or gametocytes or in the *ANKA 2.34* background parasite line. Similarly, western blot analysis using the *c57::3xha* transgenic line and the α-HA antibody confirmed expression of the PbPIMMS57::3xHA protein in the ookinete at the expected molecular weight and its absence from gametocytes (**Figure 3B**). The protein was mostly detected in the Triton insoluble fraction, indicating membrane association. No band was detected in the *c507* reference line.

To validate the ookinete expression of PbPIMMS57, the α-Pbc57 peptide antibody was utilized. In westerns, the α-Pbc57 antibody detected in ookinetes a band at about 90 kDa, close to the predicted molecular weight of PbPIMMS57. This band was not seen in MBS, gametocytes and the control *Δc57* (see below) knockout parasite line (**Figure 3C**). Fractionation assays revealed that, in ookinetes, PbPIMMS57 is mostly found in the Triton X-100 insoluble fraction with low amounts also seen in the Triton X-100 soluble fraction (**Figure 3D**), suggesting that Pbc57 is membrane associated. These results agree with the prediction of transmembrane domains in this protein. The α-Pbc57 peptide antibody did not work in IFAs.

IFAs on the developmentally compromised *c57::gfp* and *c57::3xha* transgenic lines revealed cytoplasmic localization of the PbPIMMS57::GFP and PbPIMMS57::3xHA fusion proteins with bias for the ookinete convex side, especially for the former, pointing to a possible involvement of the protein with ookinete motility or invasion machinery (**Figure 3E**). However, given that both types of fusions lead to developmentally compromised parasites, these data must be interpreted with caution.

Similar analysis in *P. falciparum* using the α-Pfc57 peptide antibody detected a clear band albeit of lower-than-expected molecular weight in western blots of midgut homogenates of *A. coluzzii* mosquitoes infected 22 h earlier with *P. falciparum* NF54 cultured gametocytes (**Figure 3F**). This in conjunction with additional low molecular weight bands could be attributed to degradation in the highly proteolytic environment of the midgut blood bolus. In IFAs, the α-Pfc57 antibody showed that PfPIMMS57 is localized in the cytoplasm of gametes/early zygotes and intermediate ookinete developmental stages (mostly stages III and IV) obtained from the midgut blood bolus at 2 and 19 hpbf, respectively, with their distribution extending to the ookinete periphery (**Figure 3G**). Although it is difficult to decipher a clear membrane distribution of the signal, apparent similarities with the Pfs25 signal in the ookinete periphery makes a membrane-associated localization of PfPIMMS57 highly probable.

#### PIMMS22 Expression and Localization

**Figure 4:**
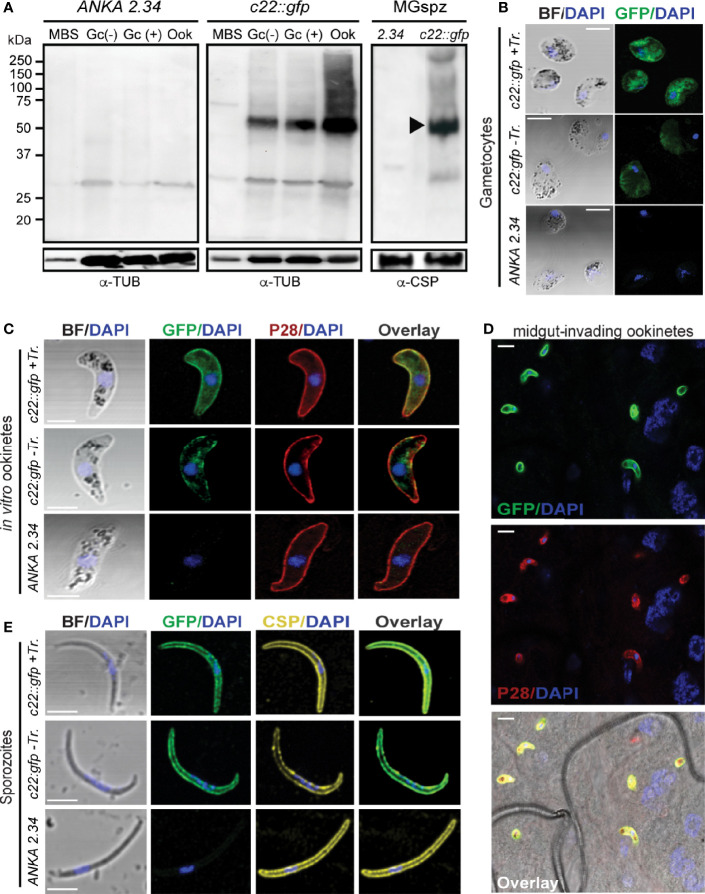
PIMMS22 protein expression and localization. **(A)** Western blot analysis using α-GFP antibody on whole cell lysates of *P. berghei c22::gfp* parasites. The PbPIMMS22::GFP fusion protein band is indicated with a black arrowhead. The *ANKA 2.34* parental parasite line was used as a negative control. Tubulin was used a loading control in all parasite stages except from midgut sporozoites where CSP was used. MBS, mixed blood stages; Gc(-), non-activated gametocytes; Gc(+), activated gametocytes; Ook, ookinetes; MgSpz, midgut sporozoites. Immunofluorescence assays of *c22::gfp* blood stage gametocytes treated or not treated with Triton X-100 (Tr.) **(B)**, *in vitro* ookinetes **(C)**, ookinetes traversing the mosquito midgut epithelium at 26 hpbf **(D)** and midgut sporozoites at 15 dpbf **(E)**, stained with α-GFP (green), ookinete surface α-P28 (red), or sporozoite surface α-PbCSP (yellow) antibodies. DNA was stained with DAPI. Staining of the *2.34 wt* parental parasite was used as a negative control. Images are de-convoluted projections of confocal stacks. BF, bright field; Scale bars in **(A–C, E)** 5 μm; Scale bars in **(D)** 10 μm.

Western blot analysis using the *c22::gfp* transgenic parasite line and α-GFP antibody revealed high levels of the PbPIMMS22::GFP fusion protein in ookinete and oocyst derived sporozoites, at the expected molecular weight of 71 kDa (**Figure 4A**). This band was not detected in the *ANKA 2.34* background parasite line. IFAs on the *c22::gfp* parasites allowed us to analyze the sub-cellular localization of the fusion protein. In Triton X-100 permeabilized blood stage gametocytes, PbPIMMS22::GFP was found to be localized on the cell periphery with the majority of the protein localizing in the cytoplasm (**Figure 4B**). Non-Triton X-100 permeabilized blood stage gametocytes are visibly less fluorescent than their Triton X-100 permeabilized counterparts. In Triton X-100 permeabilized *in vitro* ookinetes, PbPIMMS22::GFP was clearly observed on the ookinete periphery, a result that is inconsistent with the predicted absence of a signal peptide or transmembrane domain (**Figure 4C**). Additional staining on non-Triton X-100 permeabilized ookinetes show some peripheral signal albeit much weaker than its Triton X-100 permeabilized counterpart suggesting that PbPIMMS22::GFP is mainly localized on the inner surface of the ookinete and not on the plasma membrane. IFAs on *in vivo* midgut epithelium invading ookinetes at 26 hpbf also show surface localization of PbPIMMS22::GFP (**Figure 4D**). In midgut sporozoites, PbPIMMS22::GFP is found on the surface of both Triton X-100 and non-Triton X-100 permeabilized sporozoites (**Figure 4E**).

### Generation and Phenotypic Analysis of *P. berghei* Mutant Parasites

Genetically modified *c507 P. berghei* lines, designated *Δc01*, *Δc57*, and *Δc22*, were generated by replacing most of the coding regions of *PbPIMMS01*, 57, and 22 with a modified *Toxoplasma gondii* pyrimethamine resistance (TgDHFR) expression cassette, respectively (**Figures S8A–C**). Integration of the disruption cassette and generation of clonal lines was confirmed by PCR and pulse field gel electrophoresis (**Figures S8D–E**).

**Figure 5:**
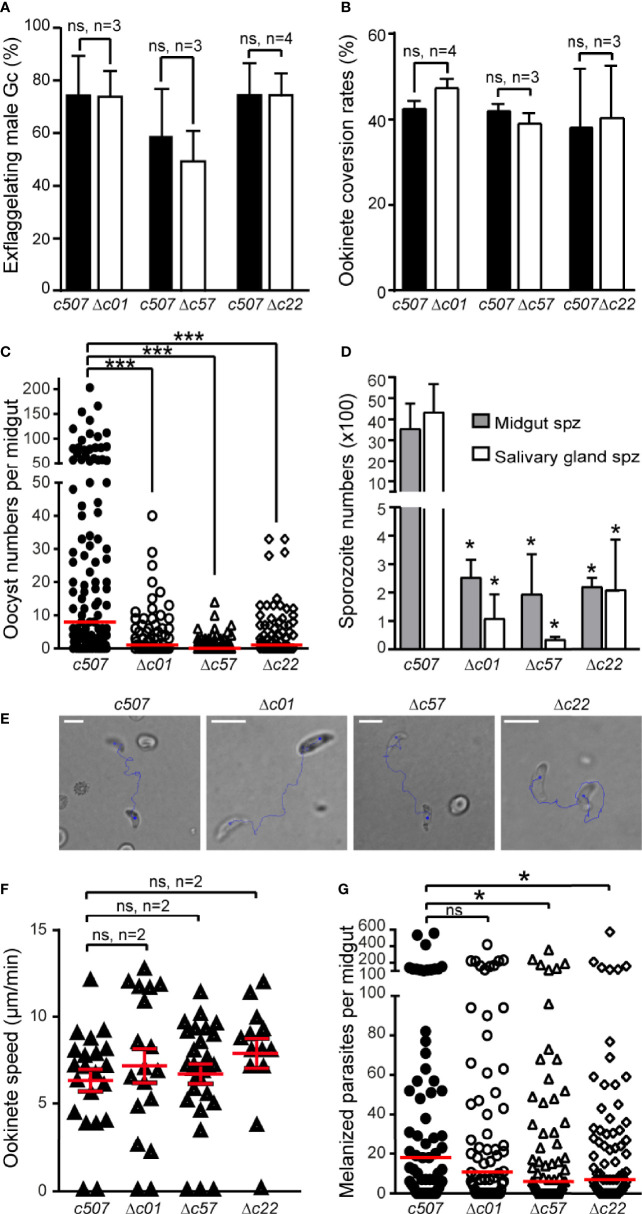
Phenotypic analysis of *P. berghei* Δc01, Δc57, and Δc22 knockout mutants. Male gametocyte activation measured as percentage of exflagellating male gametocytes **(A)** and percentage of female gamete conversion to ookinetes **(B)** of the *c507* reference and knockout parasites. ns, not significant; n, number of biological replicates. Error bars indicate SEM. **(C)** *Δc01*, *Δc57*, and *Δc22* oocyst development at 8 dpbf in *A*. *coluzzii*. Red horizontal lines indicate median. ***P < 0.0001 using Mann-Whitney test. **(D)** *Δc01*, *Δc57*, and *Δc22* midgut (MgSpz) and salivary gland sporozoite (SgSpz) numbers at 15 and 21 dpbf respectively in *A. coluzzii*. **(E)** Representative instances of *c507* reference and *Δc01*, *Δc57*, and *Δc22* knockout ookinetes from gliding motility assays. Blue lines show ookinete gliding traces captured for 2 min. Note the gliding helical motility that is characteristic of *wt* ookinete observed in these ookinetes. Scale bars, 10 μm. **(F)** Speed of *c507* reference and knockout ookinetes measured from time-lapse microscopy. ns, not significant; n, number of biological replicates. Red horizontal lines indicate mean and error bars show SEM. **(G)** Melanized ookinete numbers in *CTL4* kd *A*. *coluzzii* infected with *c507* reference and knockout parasite lines. Red lines indicate median; ns, not significant; *P < 0.05 using Mann-Whitney test.

Male gametogenesis in all three knockout parasite lines was assessed by counting exflagellation centers and found to be comparable to that of the *c507* parental line (**Figure 5A**). The macrogamete (female gamete) to ookinete conversion rates for all knockout lines were also comparable to that of the *c507* parental reference line (**Figure 5B**). These two datasets indicated that gametocyte-ookinete development is not affected in any of these knockout parasite lines.

Next, we assessed the ability of the knockout lines to complete further developmental steps in *A. coluzzii* mosquitoes that were fed on mice each infected with a knockout parasite line or the *c507* parental line. Significant decreases in the numbers of oocysts present in the mosquito midguts at 8 dpbf were observed for all the three knockout lines compared to *c507* (**Figure 5C**, **Table S3**), indicating that ookinete to oocyst development is defective all three lines.

The ability of knockout mutant parasites to produce sporozoites was assessed by counting midgut and salivary gland sporozoites 15 and 21 dpbf, respectively. Compared to the *c50*7 reference line, significant decreases in midgut and salivary gland sporozoite numbers were observed for all three knockout lines (**Figure 5D**, **Table S4**). None of the three knockout parasites could be transmitted back to mice through mosquito bites carried out 21 dpbf, in all cases leading to termination in malaria transmission (**Table S4**).

Parasites displaying normal ookinete development but showing a defect during the ookinete to oocyst developmental transition could be a result of a defect in ookinete motility and/or a midgut invasion and traversal. To investigate for motility defects, speed measurements of ookinetes were carried out. *Δc01*, *Δc57*, and *Δc22 in vitro* cultured ookinetes exhibited movements with speed not significantly different to that of the *c507 wt* ookinete (**Figures 5E, F** and **Movies S1** **–** **8**).

Next, we investigated for invasion defects by carrying out mosquito infections in *A. coluzzii* mosquitoes silenced for C-type lectin *CTL4* using RNA interference. Knockdown of *CTL4* leads to melanization of ookinetes immediately after they have traversed the midgut epithelium and been exposed to the haemocoel in the basal sub-epithelial space thereby providing a means to visualize and enumerate ookinetes that successfully traverse the midgut epithelium. The number of melanized *Δc01* parasites was not significantly different to the *c507* reference parasite line (p=0.3971) (**Figure 5G**, **Table S5**), suggesting that *Δc01* ookinetes can readily invade and traverse the midgut epithelium but are defective at the ookinete-to-oocyst transition stage resulting in the significant decrease in the oocyst numbers observed. However, compared to the *c507* parasite, significantly lower numbers of melanized *Δc57* (p=0.0337) and *Δc22* (p= 0.0487) ookinetes was observed, indicating that *Δc57* and *Δc22* ookinetes are defective in midgut invasion. However, the documented reduction in midgut invasion capacity of *Δc57* and *Δc22* ookinetes cannot fully explain the massive reduction in oocyst numbers, suggesting that like *Δc01*, *Δc57*, and *Δc22* are also defective for ookinete to oocyst transition.

The ookinete to oocyst developmental transition potential of *Δc01*, *Δc57*, and *Δc22* ookinetes was further assessed by bypassing the midgut epithelium entirely and injecting these ookinetes into the haemocoel of *A. coluzzii* mosquitoes. By skipping midgut epithelium invasion, *Δc57* ookinetes can transform to oocysts that produce salivary gland sporozoites at numbers comparable to those of the *c507* reference line (**Table 1**). These sporozoites could also be transmitted to mice in transmission experiments. In this experiment, while the mean *Δc01* salivary gland sporozoite number was significantly lower than the *c507* parasite, high *Δc01* salivary gland sporozoite numbers observed in some replicates resulted in transmission to mice while lower salivary gland sporozoite numbers resulted in no transmission suggesting that *Δc01* transmission efficiency is dependent on sporozoite numbers. For the *Δc22* parasite line, the significantly smaller number of salivary gland sporozoites produced were still not able to initiate transmission (**Table 1**).

**Table 1:** Sporozoite development and infectivity upon ookinete injection in the haemocoel.

	Salivary gland sporozoites		Infectivity to mice
Parasite	Mean	SEM	
*c507*	2,907 (3036;1888;4850;2842;2175;2650)	389	12/12 (2/2;2/2;ND;3/3;3/3;2/2)
*Δc01*	973 (582;683;492;2653;824;603)	310	3/12 (0/2;0/2;ND;2/3;1/3;0/2)
*Δc57*	3,460 (2328;6955;3864;2105;3050;2458)	680	12/12 (2/2;2/2;ND;3/3;3/3;2/2)
*Δc22*	501 (995;216;560;586;396;252)	107	0/12 (0/2;0/2;ND;0/3;0/3;0/2)

Mean salivary gland sporozoite numbers 21 days post A. coluzzii haemocoel inoculation with ookinetes, obtained from six biological replicates. Data from independent biological replicates are presented in brackets and are separated with semicolons. SEM represents the standard error of the mean. Infectivity of sporozoites was assessed by infected mosquito bite back experiments of C57/BL6 mice at day 21 post haemocoel inoculation. Parasitemia was monitored for 14 days post mosquito bite and the ratio of infected to all mice used is shown. ND, not determined.

## Discussion

To establish a successful infection in the mosquito, *Plasmodium* parasites must within 24 h of uptake into the mosquito midgut, fertilize to form ookinetes that have to invade and traverse the midgut epithelium and form oocysts on the basal side of the epithelium. The molecular processes driving the *Plasmodium* ookinete to oocyst developmental transition remain relatively under characterized. In this study, we have identified PIMMS01, PIMMS57, and PIMMS22 as being important factors for ookinete to oocyst transition and essential for malaria transmission. While gametocyte to ookinete development is not affected in the mutant knockout parasites, they all display severe defects in oocyst formation. The midgut invasion capabilities of these knockout parasites suggest that other factors come into play to block this ookinete to oocyst transitional step. The decrease in number of subsequent sporozoites eventually result in an abolishment of malaria transmission. The observed reduction in sporozoites may not be merely an effect of the oocyst defective phenotype. Since the gene knock out system used in this study is not regulatable, gene functions cannot be assessed past the point of their initial essential action. This means that additional functions of these genes past the ookinete to oocyst developmental transition in oocyst and sporozoite development and in malaria transmission cannot be ruled out. This is especially true for *PIMMS22* which shows peak transcript expression during oocyst sporozoite development and its concomitant protein is highly expressed also at this stage.

Amongst genes reported to function during the ookinete to oocyst developmental transition, an overwhelming defect before and at entry into the midgut is observed. The ookinete to oocyst defective mutants of *CTRP*, *PPKL*, *CDPK3*, *GCβ*, *DHHC3*, *OMD*, and the alveolins *IMC1 b* and *h* are defective in ookinete motility (Dessens et al., 1999; Siden-Kiamos et al., 2006; Tremp et al., 2008; Moon et al., 2009; Guttery et al., 2012; Volkmann et al., 2012; Hopp et al., 2016; Currà et al., 2019), *CHT* mutants are defective in ookinete penetration of the peritrophic matrix (Dessens et al., 2001) and *P25*, *P28*, *SOAP*, *PPLP3*, *PPLP4*, *PPLP5*, *PSOP2*, *7*, and *9* and *Enolase* mutants are defective in ookinete midgut invasion (Tomas et al., 2001; Dessens et al., 2003; Kadota et al., 2004; Ecker et al., 2007; Ecker et al., 2008; Ghosh et al., 2011; Deligianni et al., 2018). Only null mutants of *CelTOS*, *MISFIT*, *PPM5*, *AP2-O4*, *PIMMS2*, and *PIMMS43* have been identified to have a midgut invasion independent phenotype with their null mutant ookinetes able to invade the midgut epithelium but have problems in establishing an oocyst infection (Kariu et al., 2006; Bushell et al., 2009; Guttery et al., 2014; Modrzynska et al., 2017; Ukegbu et al., 2017; Ukegbu et al., 2020). Cell traversal upon midgut invasion is not compromised in *Δc01* mutant ookinetes like that observed in the oocyst formation defective *PIMMS2* and *CelTOS* mutants (Kariu et al., 2006; Ukegbu et al., 2017), as melanized *Δc01* mutant ookinetes must have traverse the midgut epithelium to reach the basal lamina where the melanization response occurs (Osta et al., 2004). In the *MISFIT* and *PPM5* mutants, the reduced number of oocysts formed are smaller and fail to complete sporulation (Bushell et al., 2009; Guttery et al., 2014). Our observations show that the small number of oocysts formed by the *Δc01*, *Δc57*, and *Δc22* parasites are morphologically normal with sizes comparable to *wt* oocysts suggesting these proteins function differently from MISFIT and PPM5.

The observation that parasite development is rescued when *Δc57* ookinetes bypass the epithelium, suggests that the viability of these ookinetes and their inherent ability to differentiate into the oocyst is not affected. This implies that the ookinete to oocyst developmental transition is only impaired when ookinetes must cross the midgut wall. This type of phenotype has been previously observed in the *SHLP1* mutant (Patzewitz et al., 2013). There, it has been suggested that developmental defects in the ookinete such as in microneme deficiency, results in a block in oocyst formation following midgut invasion which is restored if mutant ookinetes are injected into the haemocoel (Patzewitz et al., 2013). However, this may not be the case for *Δpbc57*, as ookinete microneme deficiency has been previously shown not to be essential for the ookinete transformation to oocyst (Bushell et al., 2009). Whether any ookinete morphological/developmental defects are involved with the seemingly dual phenotype of the *Δpbc57* parasite requires further investigation. Indeed, the ookinete injection experiment has shown that the reduced *Δc57* oocyst and salivary gland sporozoites obtained with the natural route of infection is a direct result of the reduced number of oocysts and not due to any additional function of PIMMS57 at the oocyst and sporozoite developmental stages.

The ookinete injection phenotypes of *Δc01* and *Δc22* indicate that the defective oocyst formation phenotypes are independent of midgut invasion suggesting that the viability of these ookinetes are overall affected *in vivo* in the mosquito. While the sporozoite numbers for *Δc01* and *Δc22* appeared to be higher than those obtained from the direct blood feeding, the observation that the sporozoite numbers produced, following injection, are on average lower than the *wt* parasite indicates that their development is still impaired. As it is impossible to accurately assess ookinete to oocyst transformation in this experiment (oocyst enumeration upon ookinete haemocoel injection is unreliable as oocysts are formed everywhere in the haemocoel-bathed tissues) (Paskewitz and Shi, 2005), it is possible that *Δc01* and *Δc22* ookinetes can transform to oocysts but the reduced sporozoite numbers are the result of impaired oocyst sporogony or defect at the transition of oocyst sporozoites to the salivary glands. As expected and has been previously observed (Churcher et al., 2017), *Δc01* salivary gland sporozoite numbers following ookinete injection significantly affects transmission. Finally, the observation that *Δc22* sporozoites produced following ookinete injection could still not be transmitted suggests putative additional functions of *PIMMS22* during this stage.

The observed impaired parasite development suggests putative roles of PIMMS01, PIMMS57, and PIMMS22 proteins in interaction with the midgut to either promote ookinete fitness or ookinete-to-oocyst differentiation. PIMMS01 and PIMMS57 are predicted to be secreted and membrane-bound, respectively, while PIMMS22 does not show any putative transmembrane domains but is clearly localized at the ookinete periphery, possibly the inner membrane complex. While the results showing that PIMMS01 and PIMMS57 are also localized at the ookinete periphery must be interpreted with caution, midgut crossing ookinetes show a clear peripheral localization of PIMMS01 and PIMMS57 has been previously also shown by others to localize on the ookinete surface (Zheng et al., 2016). All these are suggestive of a capability of these proteins to interact, directly or indirectly, with the midgut environment.

The mosquito midgut epithelium *via* effectors of the JNK pathway has been proposed to actively mark invading ookinetes for killing by the mosquito complement-like system (de Almeida Oliveira et al., 2012). The GPI-anchored ookinete surface protein Pfs47 has been suggested to protect *P. falciparum* ookinetes against attack by this system (Molina-Cruz et al., 2013), and we have previously shown that the ookinete surface protein PIMMS43 is also essential in protecting ookinetes by complement-like responses (Ukegbu et al., 2020). While PIMMS01, PIMMS57, and PIMMS22 may directly interact with the midgut, much like Pfs47 whose receptor in the midgut epithelium has been recently identified (Molina-Cruz et al., 2020) to promote ookinete survival, an alternative explanation is that loss of function of these proteins may bear a fitness cost on ookinetes. In the midgut, where oxidative stress is high due to the blood meal (Turturice et al., 2013) and *P. berghe*i infection of the midgut has been shown to exacerbate the production of reactive oxygen species (ROS) (Molina-Cruz et al., 2008), ookinetes lacking such proteins are expected to be compromised.

A clue to the possible functions of these proteins is the presence of defining signatures in their amino acid sequences. Apart from the signal peptide, PIMMS01 is devoid of any other domains that could predict its putative function. The identification of putative PP1c binding motifs in *P. falciparum* PIMMS57 suggests that it may function through PP1. In eukaryotes, PP1 is essential for cell cycle progression (Bollen et al., 2009). The exact role of PP1 in *Plasmodium* has not been deciphered yet due to its essentiality in asexual blood stages (Guttery et al., 2014). Nevertheless, the identification of several PP1c interacting proteins that can modulate the activity of this enzyme (Daher et al., 2006; Fréville et al., 2012; Hollin et al., 2016), including the gametocyte exported protein GEXP15 that is important for both blood stage development and oocyst formation (Hollin et al., 2019), could suggest additional and important roles of PP1-like activity during sexual development in the mosquito. The putative physical interaction between PfPIMMS57 and PfPP1c remains to be confirmed in functional interaction studies. The lack of PP1c binding motifs in the rest of the PIMMS57 orthologs suggest that these may function independently of PP1.

Like PIMMS01, PIMMS22 lacks any domain that could predict its putative function. Its localization and putative interacting partners could however point to the function of this protein. While we have shown PbPIMMS22 to localize at the ookinete periphery and putatively on the inner surface of the ookinete, how it achieves this without a predicted signal peptide or transmembrane domain remains to be investigated. A hypothesis is that PIMMSS22 could be interacting with other proteins located on the inner surface of the ookinete. This theory is supported by the observation that in *P. yoelii* sporozoites, PIMMS22 is found in a complex with several alveolin proteins of the subpellicular network and glideosome-associated proteins of the inner membrane complex (Muñoz et al., 2017). This interaction with the alveolin proteins could also be extended to the ookinete as some members of the SPN (subpellicular network) and IMC (inner membrane complex) are conserved and utilized across the ookinete and sporozoite stages (Morrissette and Sibley, 2002; Santos et al., 2009; Al-Khattaf et al., 2015). Proteins associated with the SPN and IMC are mostly linked to functions relating to cell motility (Tremp and Dessens, 2011; Volkmann et al., 2012; Frénal et al., 2017); however, this is not the case for PIMMS22 as no defect in ookinete motility is observed. Any putative functional interactions between PIMMS22 and proteins associated with the SPN and IMC will have to be further investigated in co-localization and pull-down experiments.

Despite the unknown exact molecular mechanisms that PIMMS01, PIMMS57, and PIMMS22 utilize to promote the ookinete to oocyst developmental transition, these proteins are good targets for the development of transmission blocking interventions. Two approaches are envisaged. First, and like the current frontline transmission blocking vaccine candidates Pfs230, Pfs48/45, and Pfs25 that target gametocyte/ookinete surface proteins, antibodies against these proteins can be generated in the human serum which, when ingested by mosquitoes together with gametocytes, interfere with the function of these proteins and block transmission to a new host (Nikolaeva et al., 2015). While this approach has been hampered by the difficulty in recombinant expression of full length and correctly folded *Plasmodium* proteins in a high throughput manner (Nikolaeva et al., 2020), the identification of small proteins that can be easily expressed such as PIMMS01 bears hopes for this approach. An alternative approach includes the creation of genetically modified mosquitoes which express single-chain antibodies that bind these proteins conferring refractoriness to infection and eventual blocking of malaria transmission (Isaacs et al., 2011; Gantz et al., 2015). These transgenes can be spread within wild mosquito populations through gene drive mechanisms (e.g. CRISPR/Cas9) leading to sustainable local malaria elimination (Carballar-Lejarazú and James, 2017).

## Data Availability Statement

The original contributions presented in the study are included in the article/**Supplementary Material**. Further inquiries can be directed to the corresponding author.

## Ethics Statement

The animal study was reviewed and approved by Animal Welfare and Ethical Review Body (AWERB), Imperial College London.

## Author Contributions

Conceptualization, DV. Methodology, CU and DV. Formal analysis, CU and DV. Investigation, CU and DV. Resources, GC and DV. Data curation, CU and DV. Writing paper, CU, GC, and DV. Supervision, DV. Project administration, GC and DV. Funding acquisition, GC and DV. All authors contributed to the article and approved the submitted version.

## Funding

The work was funded by a Wellcome Trust Investigator Award (107983/Z/15/Z) to GC, a Wellcome Trust Project grant (093587/Z/10/Z) to GC and DV, and a Bill and Melinda Gates Foundation grant (OPP1158151) to GC.

## Conflict of Interest

The authors declare that the research was conducted in the absence of any commercial or financial relationships that could be construed as a potential conflict of interest.
