# Supplementary material for: Identification of Three Novel Plasmodium Factors Involved in Ookinete to Oocyst Developmental Transition
Source: Front Cell Infect Microbiol. 2021 Mar 15;11:634273. doi: 10.3389/fcimb.2021.634273 (PMC8005625; doi:10.3389/fcimb.2021.634273)
Supplement: Supplementary file 1 [file Image_1.pdf]

Supplementary Figures

```
PfPIMMS01        M---KLLNNRFVVLCPIIILFFFLNSVVLGNNNRNNINFHETENAAKAMRKLLSGEINSI 57
PbPIMMS01        MMHFPKYSYTLCIILIFALLF----TIASSYNNEDNKNFNGTENAAKAIRKLLNGEIDTI 56
PyPIMMS01        M-YFPKYSYTLCIILILSLLF----TIASSYNNEGNKNFNETENAAKAIRKLLNGEIDTI 55
PcPIMMS01        MARFPKYSYILCIILIFALLF----TTAFSYNNGDNKNFNETENAAKAIRKLLNGEIDTI 56
PkPIMMS01        M------------YFLHNIIICPVRSCYTGRKEHEQSRICETENAARAIRKLLNGEIDTI 48
PvPIMMS01        M---KLLSASPCTLYIT-LLFVLLVHVTVGGKHMNNQDFAETEQAARAIRKLLNGEIDTI 56
Consensus                          :::        . :.  :  :  **:**:*:****.***::*

PfPIMMS01        KLDNGDELKIKLNDEKHKDSTKWD--KSYSFISNLEEEKYSQTDLFRKKQEINEANTKII 115
PbPIMMS01        KLENGDELKTKSSSNIQGD---------YSWKNNNRRL---------------------- 85
PyPIMMS01        KLENGDELKIKSSNNIQED---------YSWKNNNRQL---------------------- 84
PcPIMMS01        KLENGDELKIRSDSSIQDD---------YSWQNNNRRL---------------------- 85
PkPIMMS01        KLENGNELKIRPRDEKYDAHKRYNDETNYSFIDNSEEEN-VQTDLLRKN-ESVEVGRKI- 105
PvPIMMS01        KLENGNKLKIRSSDEKDDAHKRHNDKTNYSFIDNSEEEN-VQTNLLRKN-ETVDAGSKIV 114
Consensus        **:**::** :  ..             **: .*  .

PfPIMMS01        EDRQEFYILNNDEIENIATRFVLENNFDELYIQSFKQSLIDIIQSLNN 163
PbPIMMS01        ------------------------------------------------ 85
PyPIMMS01        ------------------------------------------------ 84
PcPIMMS01        ------------------------------------------------ 85
PkPIMMS01        DEQENFHTLDNESTESHSKQISHGNKFHESETEAFAQNLKDIIRSLNN 153
PvPIMMS01        EDQEDFYILDNESIEAIANKISMENKFHEFEAEAFAQSLKDIIRSLNN 162
Consensus
```

Figure S1. Multiple sequence alignment of *Plasmodium* PIMMS01 orthologues

Sequence alignment of PIMMS01 orthologs in six *Plasmodium* species: *P. falciparum* (PfPIMMS01; PF3D7_ 0112100), *P. berghei* (PbPIMMS01; PBANKA_0201700), *P. yoelii* (PyPIMMS01; PYYM_0204600), *P. chabaudi* (PcPIMMS01; PCHAS_0200100), *P. knowlesi* (PkPIMMS01; PKNH_0201500) and *P. vivax* (PvPIMMS01; PVX_ 081200). In the consensus sequence, dots and colons mark conserved amino acid residues with weakly and strongly similar properties, respectively, and asterisks mark amino acid residues that are identical between orthologues. Predicted signal peptides are highlighted in grey.

```
PfPIMMS57       M-GKCDFFIFL-PIFFFVLIWYVHYVLGQPSDVPNVRDLIKRFENSNESVNSNNNIFNRN 58
PbPIMMS57       MTRNLLFFIYY-HAVIFMIFWKFGNGKGGDEQKRNVKDLIRRYESINNSN---------- 49
PyPIMMS57       MKRNLLFFIYY-HAAIFMVFWKFGNGKGGNEKKKNVKDLIRRYESINNSNDLNNSSSSSS 59
PcPIMMS57       MKRNILFFIYC-HAAICIVFWKFGNGHNGNEKKRNVKDLIRRYETINNPSDLNNSSSTSA 59
PkPIMMS57       MLPKCILSIYIISCVLLLLSWKIGDVNGQLPRRGNVNELIRRFENQSTEDRQQRDSNEN- 59
PvPIMMS57       MVPKCIFSIYATFCALLLLSWNIGDVKGQPPKRGRVNDLIKWFEHGGSEDTQRRDSNEN- 59
Consensus          :  : *:     : :: * .    .      .*.:**: :*  .

PfPIMMS57       NQLERNIHYDPNNLYNIRQN--PRHENIAGRSYVSRNPNEFSSD------------IMIN 104
PbPIMMS57       ---DSNYE-DRDNAPNRRQNV-AFDDQKPSTSYANRNDDKQYGRMNMKENAKNDGNNMAN 104
PyPIMMS57       DLTDTNYE-DRDNAPNRRQNV-AFDDQQPSTSYANRNNGNQYGRMNMKGNANNDGNNMIN 117
PcPIMMS57       ESTGAPYE-NRDPIPYRGQNA-PIDDQQPSTSYANRSNESQYMRMDMPN----------- 106
PkPIMMS57       KSFESGSQ-GIEHPLNKGQNTTIYEEPQPGQSYEPARRS-SSF---------PTQKSSLN 108
PvPIMMS57       KSFQSGSQ-GWEQPLNRGQNTSIYDEQQPGTSYGPVRRSSASL---------PTEKRSFS 109
Consensus              . . :      **    .:   . **

PfPIMMS57       RNNVHDEQRVRD----------------------------------EGRE-------NN 122
PbPIMMS57       DEDYDDD-NSKSEKFNYHEPSNN----YRGNNNYMYDSNTQIP-------MYSNKSNLYS 152
PyPIMMS57       DEDYDDDDDSDSEEFNYQEPSNN----YRGNNNYMHNSNSQTP-------MYANKSYLYS 166
PcPIMMS57       TEDYDD-DDSDSDEFSYSQISNN----NRGNNNYMYDSNSQVP-------RHGNKSSLFS 154
PkPIMMS57       VDDYSDDVSDEDNDYAS-NTRNDYKRDHRIGQQHQ-----------------------RG 144
PvPIMMS57       VDDYSEDDSDEDNDYGG-NLGNDYPRDYRNDQQHQYGSSHQMRQTYDQAEEDDRDSIKHG 168
Consensus        ::  :     .                                               .

PfPIMMS57       RRSSVPNNAHTSAKYYGSDQEIMQNKPVNNLHLGKSLDDFYNNLGVSSDDFEDDEQVNYN 182
PbPIMMS57       SNFRDPNENHSSENYGD-----QYSPQNSPNYLDRSFDDFRNKLGLSSEDFSRETETYSN 207
PyPIMMS57       SNFRDPNENQPSENYDD-----QYSPQNNPPYLGRSFDNFRNRLGLNTEDFSRGTETYSN 221
PcPIMMS57       NNRRDPNENSPSGKYGD-----QDSSQNNAPYLGRSFDNFRNKLGLSSEDFSRDSEYYSN 209
PkPIMMS57       NRDGFPDEEPPRREEGG-----RMT-PKGNTYLGNDFEHFNKRLGLSMDDYMSDTEDDSF 198
PvPIMMS57       NRDGYPDEGPPRREEGG-----RMT-PNGNVYLGKDFEHFNKRLGLSMDDYMSDKEDDSM 222
Consensus        .   *::     :  .       .   .  :*...::.* :.**:. :*:    :

PfPIMMS57       END----AFSSFSHNNQNDDTTLLYDEFEYVDSNNYTRNGSSSSSNNNNNNNNNIGNNGK 238
PbPIMMS57       TTNKRNT-------GLDNDLNSFVTDDIQYTKYESTENP------INSSNQRRDSGMR-- 252
PyPIMMS57       TTNKRNT-------GLNNDSDSFITDDIQYTKYESSENP------INSSRQRHDSGMR-- 266
PcPIMMS57       TNNRRNT-------GLNDEFNTPIADDIQYTKYGNSENS------NNSTRNRRDSGMR-- 254
PkPIMMS57       DSSDNSSDLPRNKLNAHDTDSSSIYEDIQFVRHRDNRQP------NDDVQFSRTDGRR-- 250
PvPIMMS57       GSSNNSSDLRSNESNAYDADQSSIYEDIQFVKRRNHRQP------NKHVQLSKTDGRR-- 274
Consensus        ..           .  :   : : ::::.    .  .        .  .  .  * .

PfPIMMS57       ITYNENDVSYNDSNNGNSTLSNIHRDNKIKPNLS-----SLYGNKTFNNYSNST------ 287
PbPIMMS57       ---QSPNNEYNDRNNNNG--NNRNSNMYINPNEYDDTYTNSYGYNSQNKSINSSYVMRYG 307
PyPIMMS57       ---QSPNNEYNDRNNN-S--NNRNSNMYINTDEDDDSYSNPYGYNNQNQGPNASQVMRYR 320
PcPIMMS57       ---QSPSNEYNNRNNN-----NINSNTYNNPDEDDDSYFNPYGYNSQSQSSSSSQLMEHG 306
PkPIMMS57       ---NAM---YNRSDNGAEESPPRESKRV--NNLSSDVYDYAYGRNGSNMSEPAN-LYSYP 301
PvPIMMS57       ---NAI---YRSSGSDEEAPHSSESEIV--RDPSSYVYDYAYGRNGPNRSAPLN-LYSYP 325
Consensus          :     *.  ...       . .     :         ** :  .     .

PfPIMMS57       FNDVSNNYNRDDEITPFNNNRNVYVPSEYSNPLD-AYVHPNNQQFSTQQYGNNMEEMNKL 346
PbPIMMS57       LNGGNDNIDQ-Q--------------TIYSNP--IDYTSSNN------NYSSSFGGNIKP 344
PyPIMMS57       LNSDDDSIDQ-Q--------------SIYSNP--IDDISTSD------NYSLNFGGKKKS 357
PcPIMMS57       LNSGSGSIDQ-R--------------SVYSNP--LEYMSPNV------DYRPSFEGRIKP 343
PkPIMMS57       ----EDDISQ-RTIPPIENRK---SRNRSSSPLQDDYSIPSD------TYRNSYDSE--- 344
PvPIMMS57       ----EEDFNR-RTSPPVGSKK---SMSSSSSPLQDDYIKPLD------SYSDSHDSM--- 368
Consensus           . . .:                .  *.*                 *  .
```

```
PfPIMMS57   RRKVNFAPRDIYTVNNSL-------------------DG---HSINNNTPLNNMD-AGPS 383
PbPIMMS57   -RNIQHIENDNSSILNSQRDYEDSDRNS-S--RY----GDNRSNRQNKSPIRNID----- 391
PyPIMMS57   -RSIQHIENDNSSILNSQRNYEDIDNNS-S--RN----GDNRSKIQNKPSIRIFD----- 404
PcPIMMS57   -RNIQPIEDDNYNMLNSPRHYEDIDSTS-S--GN----NSNRSNSRNKPPIRSVD----- 390
PkPIMMS57   --------DHIYSVIPGEEQYEDEINQRRTRQGYSSLGNGARPDRNEILPVRGIKKSASS 396
PvPIMMS57   --------NHLYDVIPGEEQYEDEINRRRTRPRYNSFDNAVHLDRSELLPVRGVKKSASS 420
Consensus            .   :  .                     .    .  :   :. ..

PfPIMMS57   YMTEGNKNVNKNININTNTNTNTNTNTNTNTNTNTNTNTNTNKSKKRSDSNFFSKNKKNNSA 443
PbPIMMS57   ---RGNKGDA-------------------------------------QNRSKRVAHANSI 411
PyPIMMS57   ---RGNKGDA-------------------------------------QNKSKRLSYSNSN 424
PcPIMMS57   ---RGSKGDA-------------------------------------QNRSSRMSRSNSD 410
PkPIMMS57   YHPEGRESKT-------------------------------------DGSLKKVRKNNKE 419
PvPIMMS57   YQPEGGKGKM-------------------------------------DNFFNKFRKSKRV 443
Consensus      .* :.                                       ..  .:    :

PfPIMMS57   STNE------L-SRYKDVSKKIFKDNMYNRYENKNYPQSVYVFNEYQNNFFFNKILKRS- 495
PbPIMMS57   PI--------LHRKYDEERQ----PTAHSTITNKILASYIDIYTAQQRNIFYNKNTRVE- 458
PyPIMMS57   PI--------LNKNYE-PRQ----PITRNSTSNKILASYIDIYTAQQRNIFYNKNTRVE- 470
PcPIMMS57   PT--------VNKSYGEARQ----SAPRRAQTNKILESYIETYITHQRNIFYNKIIRVE- 457
PkPIMMS57   D-----------SSTVMHTQ----QGSKNDRNVSKPLSIITLFSTYQRNLFLNKYSKSSG 464
PvPIMMS57   DMPKGVSPASQGVSNSAHTQ----RGPKDGRQGSKSPSVLTLFTTYQRDLFLRKYSKVGG 499
Consensus                      :             .   . :  :   *.::* .*  :

PfPIMMS57   -----------SSNSIKQIFQKNTQHKNTNNNVSVTSELQVATQHCISTNKNKLTKPVIL 544
PbPIMMS57   ------------KSPIKNFFKMS---NSNDKIISVTEELLNSSKQCIIKNINKLTNEVLL 503
PyPIMMS57   ------------KSPIKKFFKMN---NSNDKIISVTEELLNSSKQCIVNNINKLTNDVLL 515
PcPIMMS57   ------------RSPVKQFFKMR---NSNDKMISVTEELLNSSKQCIVKNINKLTRDVLL 502
PkPIMMS57   KVGGNVNAN--GRTGVKRLFSFSR--SGKDSGLSIMGELQTAVEQCIVKNSNKLSRLVLM 520
PvPIMMS57   GSGGMGGSNASSSRGVKRL--FSR--SGKDDGQSITGELQTAAEQCIAKNRSKLSRPVLM 555
Consensus                  :*.:       ...:.  *:  **  : ::** .* .**:. *::

PfPIMMS57   KYIALNNKKYLNKYEYAMSYIHFTCNNKNNKKSNMCLDIKPMLYTENDKNISDMINRLPN 604
PbPIMMS57   KNLALNNTNMIKNYEQAVSYVVFNSKIKN---RESHLDIKSVPYNYSEAYVENSSVILPN 560
PyPIMMS57   KNLALNNTNMIKNYEQAVSYIVFHSKIKN---KESHLDIKSVPYKYSELHVENSSTILPN 572
PcPIMMS57   KNLALNNLNMIKNYEQTVSYIVVNSKYKN---TELHLNIKPVPYKYSEVHVENSSTMLPN 559
PkPIMMS57   KNLAFNDSKLLENYEYAVSYIRNNCNNG----SATCLDIRPMVYREDDPDASSIVTSLPN 576
PvPIMMS57   KNLAFNDPKLLKNYEYAVSYISDNCKNG----NAACLDIRPMIYREDDPDLASIVTSLPN 611
Consensus   * :*:*: : :::** ::**:   .:          *:*: : *  .:    .    ***

PfPIMMS57   VYILNTIEYILTDPKTSSSYKSLIKNNIKNHHLTVTDIVLILSNNYFKN-INSILVNHYI 663
PbPIMMS57   MYILNTYEFMLSNQRICGKFRTIIKNKKRDNNLKPVDVVLMLSFDYIKN-MNPILVSNLM 619
PyPIMMS57   MYILNTYEFMLSNQRICGRFRTIIKNKKRDNNLKPVDLVLMLSLDYIKN-MNPTLVSNLI 631
PcPIMMS57   MYILNTYEFMLSNQRMCSKFRTILKNKKRDNNLKPADVVLMLSFDYIKN-VSPRFVSNLM 618
PkPIMMS57   IYILSTYEFLLTNLRMCGPLRTMVKNRVKENKLTPTDIVLLLSGAYFKSWMNNTLVKHLI 636
PvPIMMS57   IYILSTYEFLLTNLRMCGWLRTMVKNRVKENKLTPTDIVLLLSGGYFKSWVNNTLVKHLI 671
Consensus   :***.* *::*:: : ..  ::::**. ::::*. .*:**:**  *:*. :.  :*.: :

PfPIMMS57   SFLNTKNETYLLKYYLSALISFVPFIKPAMKIYYDQR-YINLSLYTLNLEIAKVFNGLLN 722
PbPIMMS57   QFIKTKQSIYMKKYYFNIMVALIPFIKPALKIYYGEKIYKDLTTYKIQSEIKNIFDELLL 679
PyPIMMS57   KFIKTKQSIYMKKYYFNTMVALIPFIKPALKIYYGESIYKDLTTYKMQSEIKNIFDELLL 691
PcPIMMS57   QFIKTKQGIYMKKYYFDTMMALIPFIKPALKIYYGEKMYNDLTTYKIQSEIKSIFDELLL 678
PkPIMMS57   SFLKTKRVVYLEKYFLAVLISLSSFIKPALKIYFGER-YAKVSAHNLDAEVNKMIPEMLG 695
PvPIMMS57   AFLTTKRVIHLEKYFLAVLISFAPFIKPALKMYFGER-YAKLSAYSLDVEVNKMVGEMLG 730
Consensus    *:.**.  :: **::  ::::  *****:*:*:.:  * .:: :.:: *: .:.  :*
```

```
PfPIMMS57        VILKWSQAFKNENKHITNQIVIQMYKNLSSFSHNRIPNLGHMFKSLEVILRYQRIYNDIK 782
PbPIMMS57        ISIKWAQAFKDKYSATDNKVILDMHRTISEFSTSKTRTLSKTFVSLENILYKELITSDMI 739
PyPIMMS57        ISIKWAQAFKDKYSANDNKIILDMHQTISKFSTSKTRTLSKTFVSLENILYKELITNDIV 751
PcPIMMS57        ISLKWAQAFKDKYSSADNKIILDMHRTISEFSTSKTKTLSTTFVSLENILYKELISNDMI 738
PkPIMMS57        AALRWTQAFQEKFSDESNRILLNVHKTLASYSKERNGRLSRKFRTLEALLLRQKVASDVV 755
PvPIMMS57        VALRWTQAFQEKFSDESNRVLLQVHRRLAAYSKSRNGGLTGKFRTLQELLLRQKVASDVT 790
Consensus          ::*:***::: .   *:::::::: :: :* .:   *   * :*: :*  : : .*:

PfPIMMS57        KGKEDEYEVMIGYFTKYVRNVYAKTITS------- 810
PbPIMMS57        DNKDDNQKVLISYIVKCFRNIYYTTFYMMSNKRTY 774
PyPIMMS57        DNKDDNQKVLISYIVKCFRNIYYSAFYLMGNKRTY 786
PcPIMMS57        NNKDENQKVLISYIMKCFRNIYYSAFYIMNNKRN- 772
PkPIMMS57        SNQDEGYRHLIQQILKYVRNVHSTVFE-------- 782
PvPIMMS57        SNQDQGYRLLIQHVLKYVQNVHLTVLE-------- 817
Consensus        ..:::  . :*  . * .:*:: ..:
```

Figure S2. Multiple sequence alignment of *Plasmodium PIMMS57* orthologues

Sequence alignment of PIMMS57 orthologs in six Plasmodium species: *P. falciparum* (PfPIMMS57; PF3D7_1244500), *P. berghei* (PbPIMMS57; PBANKA_1457700), *P. yoelii* (PyPIMMS57; PYYM_1461700), *P. chabaudi* (PcPIMMS57; PCHAS_1460000), *P. knowlesi* (PkPIMMS57; PKNH_1463700) and *P. vivax* (PvPIMMS57; PVX_101120). In the consensus sequence, dots and colons mark conserved amino acid residues with weakly and strongly similar properties, respectively, and asterisks mark amino acid residues that are identical between orthologues. Predicted signal peptides are highlighted in grey and predicted transmembrane domains are shown with red fonts, respectively. Predicted *P. falciparum* serine/threonine protein phosphatase type I catalytic (PfPP1c) binding motifs are shown in boxes.

```
PfPIMMS22      MTNIIECTFKTPPDNAKTPDNAVIWNQFQYCDEKGWYSLSNHDEIALRPTTFNDKRIKFL 60
PbPIMMS22      MTNVVECTFKTPPETAKAPDNAVIWNRFQYCDEKGWYSLSNHDEITLRPTIFNDGRIKFL 60
PyPIMMS22      MTNVVECTFKAPPETAKAPDNAVIWNRFQYCDEKGWYSLSNHEEITLRPTIFNDGRIKFL 60
PcPIMMS22      MTNVVECTFKIPPETAKAPDNAVIWNRFQYCDEKGWYSLSNHEEITLRPTIFNDDRIKFL 60
PkPIMMS22      MTNVVECTFKTPPETAKAPENAVIWNAFQYCDEKGWYSLTNHDEIMLRPTAFSDGRIKFL 60
PvPIMMS22      MTNVVECTFKTPPETAKAPDNAIIWNSFQYCDEKGWYSLTNHDEIMLRPTAFSDGRIKFL 60
Consensus      ***::***** **:.**:*:**:*** ************:**:** **** *.* *****

PfPIMMS22      VQLPEIPSEFESILSGRYDAKAWGKEDCYVVIEGEKDVHIRLPGFKEKINYNHTERFPTF 120
PbPIMMS22      PQLDTIPEEFESVLCGKYDAKAWGKDDCNLVIEGEKDVHISLPGLKEKINYNHKERFPTF 120
PyPIMMS22      PQLDTIPEEFESVLCGKYDAKAWGKDDCNVVIEGEKDVHISLPGLKEKINYNHKERFPTF 120
PcPIMMS22      PQLDTIPEEFESVLCGKYDAKAWGKDDCNVVIEGEKDVHISLPGIKEKINYNHKERFPTF 120
PkPIMMS22      PQLEKIPDEFESVLCGKYDAKSWGKDDCNIVIEGDKDVHISLPGLQEKINYNHRERFPTF 120
PvPIMMS22      PQLEKIPEEFESVLCGKYDAKAWGKDDCNIVIEGDKDVHISLPGLQEKINYNHRERFPTF 120
Consensus       **  **.****:*.*:****:***:** :****:***** ***::******* ******

PfPIMMS22      LKNWKIIVSILNEHVTLIRINAETALIININEKKNVTVKSVDFNNGFLCVNPHTNLAIAY 180
PbPIMMS22      LKNSKIIVSLLNENLTVIRINLETALLICINEKKSVIVKSINFNNGFACVNPYSNLAITY 180
PyPIMMS22      LKNSKIVVSLLNENLTVIRINIETGLLISINEKKSVIVKSINFNNGFACVNPYSNLAIAY 180
PcPIMMS22      LKNSKIVVSLLNENITVIRINIETALLICINEKKNVIVKSINFNKGFACVNPYSNLAIAY 180
PkPIMMS22      LKNWKIIVGMLNEHITVIRINTETAIIISISEKKNVTVKCVDFNNGFLCVNPHTNLAIAY 180
PvPIMMS22      LKNWKIIVGMLNEHITVIRINTETAIIVSINEKSNVTVKCVNFNNGFLCVNPHTNLAIAY 180
Consensus      *** **:*.:***::*:**** **.::: *.**..* **.::**:** ****::****:*

PfPIMMS22      GDFALSSLKKCELIQNIPHEGGKWGFFTHLFKWGHIIIPKELEIKLPSPGLKLIGKKIDT 240
PbPIMMS22      GGFAFNDLKKCEIVPTITHSGCEWAFFVHLFKWGHIVIPKDLELKIPSSGLKLIGKKVDT 240
PyPIMMS22      GGFAFNDLKKCEIVPTITHSGCEWAFFVHLFKWGHIIIPKDLELKIPSSGLKLIGKKVDT 240
PcPIMMS22      GGFAFNDLKKCEVVPSITHSGCEWAFFVHLFKWGHIIIPKDIELKIPSPGLKLIGKRVDT 240
PkPIMMS22      GGFALSELKKCELVPSITHEGAEWGFFVHLFKWGHIIIPKDIEIKLPSPGLKLIGKKIDT 240
PvPIMMS22      GDFALSELKKCELVPNITHEGAEWGFFVHLFKWGHIIIPKDIEIKLPSPGLKLIGKKIDT 240
Consensus      *.**:..*****:: .* *.* :*.**.********:***::*:*:** *******::**

PfPIMMS22      LAIVSIPPNIHIHVKLDGPKCIRKLEYGQDYNITAIKSSESDVDIYILFDGHLLKYEFSF 300
PbPIMMS22      IAIVSLPPNIQIHVKIDGPKCIRKVEYGQDYNITAIKSSESDIDIYVLFDGQLLKYEFSY 300
PyPIMMS22      IAIISLPPNIQIHVKIDGPKCIRKVEYGQDYNITAIKSSESDIDIYVLFDGQLLKYEFSY 300
PcPIMMS22      IAIISLPPNIQIHVKIDGPKCVRKVEYGQDYNITAIKSSESDIDIYVLFDGQLLKYEFSY 300
PkPIMMS22      VAIISLPPNIYIHVKIDGPKCIRKLEYGQDYSITAIKSSESDIDIYVLFDGQLIKYEFSF 300
PvPIMMS22      VAIISLPPNIYIHVKIDGPKCIRKLEYGQDYSITAIKSSESDIDIYLLFDGQLIKYEFSF 300
Consensus      :**:*:**** ****:*****:**:******.**********:***:****:*:*****:

PfPIMMS22      DIRLNKPEKGRSLHSAKLKCINKSKEVTSFIFQETKNCKILLGSNCPSDNLGHLLNSQTI 360
PbPIMMS22      DTRLNKEGKGKSINHAKLKCTSKSKEVSTFIFQESPNCKVLLGSNCPTDNLGHMLCNQTI 360
PyPIMMS22      DTRLNKEGKGKSIHHAKLKCISKSKEVSTFVFQESQNCKVLLGSNCPTDNLGHMLCNQTI 360
PcPIMMS22      DTRLNKEGKGKSIHNAKLKCISKSKEVSTFVFQESPNCKVLLGSNCPTDNLGHMLCNQTI 360
PkPIMMS22      DTRLNKVGKGRSINCAKLKCTNKSKEVTSFIFQPTANSKLLLDSNCPTDNMGHLLCNQTM 360
PvPIMMS22      DTRLNKVGKGRSINYAKLKCTNKSKEVTSFVFQATANSKLLLDSNCPTDNMGHLLCNQTI 360
Consensus      * ****  **:*:: ***** .*****::*:** : *.*:**.****:**:**:* .**:
```

```
PfPIMMS22        AIFDAEIGEYLSHPQGLQLTSVFNTLSYPLDKE 393
PbPIMMS22        SIFDAEIGEYQSHPQGLLLTEAFEKLSYPVENA 393
PyPIMMS22        SIFDAEIGEYQSHPQGLLLTEVFEKLSYPVENA 393
PcPIMMS22        SIFDAEIGEYQSHPQGILLTEVFEKLSYPVENA 393
PkPIMMS22        SVFDAETGEYLSHPQGLKLTEVFNSLSYPPEE- 392
PvPIMMS22        SVFDAETGEYLSHPQGLQLTEVFNTLSYPPEKE 393
Consensus        ::**** *** *****: **..*:.**** ::
```

Figure S3. Multiple sequence alignment of *Plasmodium PIMMS22* orthologues

Sequence alignment of PIMMS22 orthologues in six Plasmodium species: *P. falciparum* (PfPIMMS22; PF3D7_0814600), *P. berghei* (PbPIMMS22; PBANKA_1422900), *P. yoelii* (PyPIMMS22; PY17X_1424900), *P. chabaudi* (PcPIMMS22; PCHAS_1424700), *P. knowlesi* (PkPIMMS22; PKNH_1425600) and *P. vivax* (PvPIMMS22; PVX_123040). In the consensus sequence, dots and colons mark conserved amino acid residues with weakly and strongly similar properties, respectively, and asterisks mark amino acid residues that are identical between orthologues.

```
PfPIMMS22      ---MTNIIECTFKTPPDNAKTPDNAVIWNQFQYCDEKGWYSLSNHDEIALRPTTFNDKRI 57
PbPIMMS22      ---MTNVVECTFKTPPETAKAPDNAVIWNRFQYCDEKGWYSLSNHDEITLRPTIFNDGRI 57
PyPIMMS22      ---MTNVVECTFKAPPETAKAPDNAVIWNRFQYCDEKGWYSLSNHEEITLRPTIFNDGRI 57
PcPIMMS22      ---MTNVVECTFKIPPETAKAPDNAVIWNRFQYCDEKGWYSLSNHEEITLRPTIFNDDRI 57
PkPIMMS22      ---MTNVVECTFKTPPETAKAPENAVIWNAFQYCDEKGWYSLTNHDEIMLRPTAFSDGRI 57
PvPIMMS22      ---MTNVVECTFKTPPETAKAPDNAIIWNSFQYCDEKGWYSLTNHDEIMLRPTAFSDGRI 57
T.gondii S15   ---MERQATCRYDPLVEVP-LPPGIVIWTQHQYYDGAGWLALPDREKLELKPTRWSDGRL 56
N.caninum S15  ---MERLSTCRYDPLTEVP-LPPGIVLWTPHQYYDGAGWLALPEHEKLQMKPTRWADGRL 56
E.tenella S15  MEAVNKLCSCRFEPLVEVP-VPEKAVVWTSKNYYDGSGWVGLADGEKLSLKPTLFADNRL 59
Consensus         : .   * :.   : .  *   ::*.  :* *  ** .*.: ::: ::** : * *:

PfPIMMS22      KFLVQLPEIPSEFESILSGRYDAKAWGKEDCYVVIEGEKDVHIRLPGFKEKINYNHTERF 117
PbPIMMS22      KFLPQLDTIPEEFESVLCGKYDAKAWGKDDCNLVIEGEKDVHISLPGLKEKINYNHKERF 117
PyPIMMS22      KFLPQLDTIPEEFESVLCGKYDAKAWGKDDCNVVIEGEKDVHISLPGLKEKINYNHKERF 117
PcPIMMS22      KFLPQLDTIPEEFESVLCGKYDAKAWGKDDCNVVIEGEKDVHISLPGIKEKINYNHKERF 117
PkPIMMS22      KFLPQLEKIPDEFESVLCGKYDAKSWGKDDCNIVIEGDKDVHISLPGLQEKINYNHRERF 117
PvPIMMS22      KFLPQLEKIPEEFESVLCGKYDAKAWGKDDCNIVIEGDKDVHISLPGLQEKINYNHRERF 117
T.gondii S15   RFLDPIDELPEPFKAVQSGKFDVKCWKRGDCKLGIEGDKTVFLKSPISPDVAVYVHAERL 116
N.caninum S15  RFLDPIDELPEPFKAVQSGKFDVKCWRRGDCKLGIEGDRTVFLKAPISPEVAVYVHADRL 116
E.tenella S15  LFLEPVEGVCEAFSSVQSGKYDVKCWSKLGCHLGIEGDQNVFLSTPKLKEVAVYSHPERL 119
Consensus       **  :  : . *.:: .*::*.*.* : .* : ***:: *.:  *   :   * * :*:

PfPIMMS22      PTFLKNWKIIVSILNEHVTLIRINAETALIININEKKNVTVKSVDFNNGFLCVNPHTNLA 177
PbPIMMS22      PTFLKNSKIIVSLLNENLTVIRINLETALLICINEKKSVIVKSINFNNGFACVNPYSNLA 177
PyPIMMS22      PTFLKNSKIVVSLLNENLTVIRINIETGLLISINEKKSVIVKSINFNNGFACVNPYSNLA 177
PcPIMMS22      PTFLKNSKIVVSLLNENITVIRINIETALLICINEKKNVIVKSINFNKGFACVNPYSNLA 177
PkPIMMS22      PTFLKNWKIIVGMLNEHITVIRINTETAIIISISEKKNVTVKCVDFNNGFLCVNPHTNLA 177
PvPIMMS22      PTFLKNWKIIVGMLNEHITVIRINTETAIIVSINEKSNVTVKCVNFNNGFLCVNPHTNLA 177
T.gondii S15   PTFPKSWKPLVFILNQSLAMFRLTENLCLLVVAEKDKTMNISCVDYNGGFACTHPSTNMV 176
N.caninum S15  PAFPKTWKPLVFILNQSVAMFRLTENLCLVLVVENDKTMNISCVDYNGGFACTHPKTNMV 176
E.tenella S15  PAFPKSWKPLLFTLNSSVITFRLTDTLCLVVTIDESKTTKVHCVDYNGGFALSHPASDSA 179
Consensus      *:* *. * ::  **. :  :*:.    :::  .:...  : .:::* **   :* :: .

PfPIMMS22      IAYGDFALS---SLKKCELIQNIPHEGGKWGFFTHLFKWGHIIIPKELEIKLPS--PGLK 232
PbPIMMS22      ITYGGFAFN---DLKKCEIVPTITHSGCEWAFFVHLFKWGHIVIPKDLELKIPS--SGLK 232
PyPIMMS22      IAYGGFAFN---DLKKCEIVPTITHSGCEWAFFVHLFKWGHIIIPKDLELKIPS--SGLK 232
PcPIMMS22      IAYGGFAFN---DLKKCEVVPSITHSGCEWAFFVHLFKWGHIIIPKDIELKIPS--PGLK 232
PkPIMMS22      IAYGGFALS---ELKKCELVPSITHEGAEWGFFVHLFKWGHIIIPKDIEIKLPS--PGLK 232
PvPIMMS22      IAYGDFALS---ELKKCELVPNITHEGAEWGFFVHLFKWGHIIIPKDIEIKLPS--PGLK 232
T.gondii S15   VAYGSYVLKNFEKLPSCQAIPKMLTASGDWGFFVQFYPWGFFFIPKSVELTRPQAVLGAV 236
N.caninum S15  VAYGSYIVKTFEKLSNCAAIPKMVTASGDWGFFVQFYPWGFFFIPKSLELTRPQAVLGAV 236
E.tenella S15  LAYGSLAVKGFEALPHCEVIPRVTSAAGEWGFFVQLFQWGSFVVPKAVDLTRPSSILG-M 238
Consensus      ::**.  ..    *  *  :  :   . .*.**.::: ** :.:** :::. *.   *

PfPIMMS22      LIGKKIDTLAIVSIPPNIHIHVKLDGP-KCIRKLEYGQDYNITAIKSSESDVDIYILFDG 291
PbPIMMS22      LIGKKVDTIAIVSLPPNIQIHVKIDGP-KCIRKVEYGQDYNITAIKSSESDIDIYVLFDG 291
PyPIMMS22      LIGKKVDTIAIISLPPNIQIHVKIDGP-KCIRKVEYGQDYNITAIKSSESDIDIYVLFDG 291
PcPIMMS22      LIGKRVDTIAIISLPPNIQIHVKIDGP-KCVRKVEYGQDYNITAIKSSESDIDIYVLFDG 291
PkPIMMS22      LIGKKIDTVAIISLPPNIYIHVKIDGP-KCIRKLEYGQDYSITAIKSSESDIDIYVLFDG 291
PvPIMMS22      LIGKKIDTVAIISLPPNIYIHVKIDGP-KCIRKLEYGQDYSITAIKSSESDIDIYLLFDG 291
T.gondii S15   GMGKKVDTIGLVFHPPNMFINVKLDIPAKTTRALQFGKDFQVTAKKTSETDIDVFLVIDG 296
N.caninum S15  GMGKKVDTIGLVFHPPNMFINVKLDLPAKTTRALQFGKDFQVTAKKTSETDIEIFLIIDG 296
E.tenella S15  GLGKKVDCLGVLLLPPNLVVMVHLETP-KVQRSLQHGKDYLLTAVKTSETDLDIFLVMDG 297
Consensus       :**::* :.::  ***: : *::: * *  * ::.*:*: :** *:**:*:::::::**

PfPIMMS22      HLLKYEFSFDIRLNKPEKGRSLHSAKLKCIN----KSKEVTSFIFQETKNCKILLGSNCP 347
PbPIMMS22      QLLKYEFSYDTRLNKEGKGKSINHAKLKCTS----KSKEVSTFIFQESPNCKVLLGSNCP 347
PyPIMMS22      QLLKYEFSYDTRLNKEGKGKSIHHAKLKCIS----KSKEVSTFVFQESQNCKVLLGSNCP 347
PcPIMMS22      QLLKYEFSYDTRLNKEGKGKSIHNAKLKCIS----KSKEVSTFVFQESPNCKVLLGSNCP 347
PkPIMMS22      QLIKYEFSFDTRLNKVGKGRSINCAKLKCTN----KSKEVTSFIFQPTANSKLLLDSNCP 347
PvPIMMS22      QLIKYEFSFDTRLNKVGKGRSINYAKLKCTN----KSKEVTSFVFQATANSKLLLDSNCP 347
T.gondii S15   QLAKYNYSFDIRINKPERPKHTDNIHFKCSCDAEEKKKPDPKFKLSACKDSVILLEQGCP 356
N.caninum S15  QLAKYNYSFDIRNNKPERPKHTDNIHFKCTCDAEEKKKPEPRFKLTACKDSVVLLEQGCP 356
E.tenella S15  QLSVFNYSFDLRLNRPNKPRHLDNAAFKGSLELDDKKK-CSRFIFQNTKNSKVLVPQGCP 356
Consensus      :*  :::*:* * *:  : :  .   :*       *.*  . * :    :. :*: ..**
```

```
PfPIMMS22      SDNLG-HLLNSQTIAIFDAEIGEYLSHPQGLQLTSVFNTLSYPLDKE 393
PbPIMMS22      TDNLG-HMLCNQTISIFDAEIGEYQSHPQGLLLTEAFEKLSYPVENA 393
PyPIMMS22      TDNLG-HMLCNQTISIFDAEIGEYQSHPQGLLLTEVFEKLSYPVENA 393
PcPIMMS22      TDNLG-HMLCNQTISIFDAEIGEYQSHPQGILLTEVFEKLSYPVENA 393
PkPIMMS22      TDNMG-HLLCNQTMSVFDAETGEYLSHPQGLKLTEVFNSLSYPPEE- 392
PvPIMMS22      TDNMG-HLLCNQTISVFDAETGEYLSHPQGLQLTEVFNTLSYPPEKE 393
T.gondii S15   SGNPDEQLVSEQLIACFDAEVCLYSTHPPALKLCDAFTDVAIRE--- 400
N.caninum S15  CGNPDEQLVSEQVIAFFDAEVCLYYTHPPALKLCDAFTDVAIRE--- 400
E.tenella S15  LSEGD-HLVSSSLIAVFDAEICMYLTHPPALKLCSAFNTVALPVD-- 400
Consensus       .: . ::: .. :: ****   * :** .: * ..*  ::
```

Figure S4. Multiple sequence alignment of *Plasmodium* PIMMS22 and homologues in other apicomplexans

Sequence alignment of PIMMS22 orthologues in six *Plasmodium* species and homologs in other apicomplexan parasites. UniProt ID's- *T. gond*ii S15 (CEL77905.1), *N. caninum* S15 (XP_003885565.1) and *E. tenella* S15 (XP_013230639.1). In the consensus sequence, dots and colons mark conserved amino acid residues with weakly and strongly similar properties, respectively, and asterisks mark amino acid residues that are identical between orthologues.

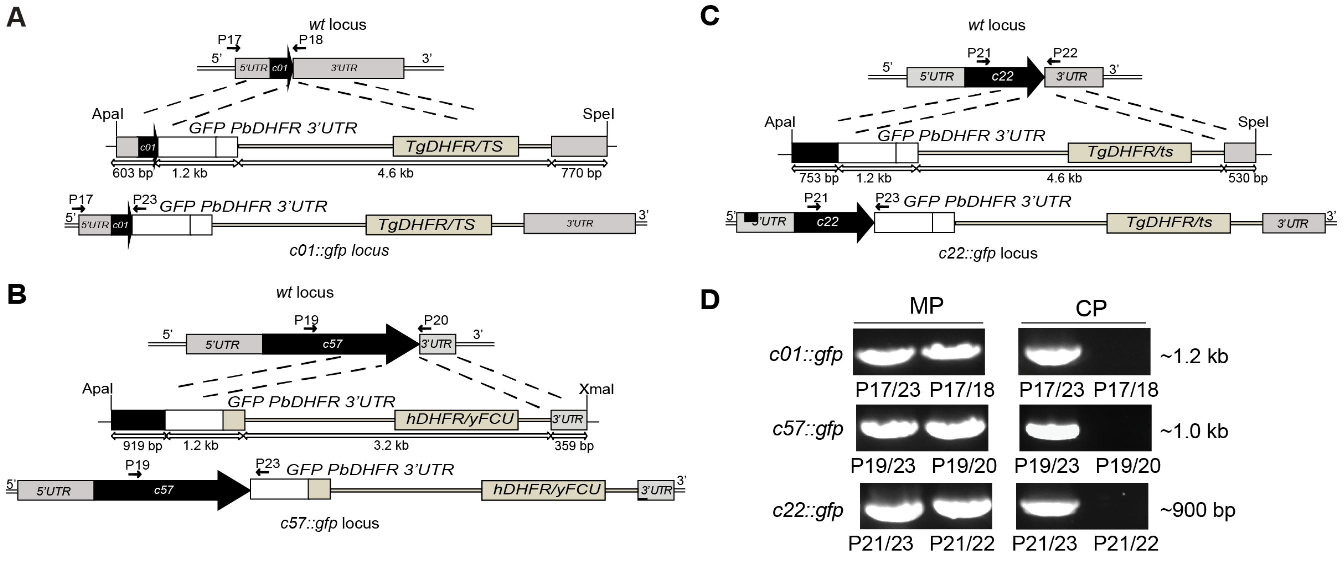

Figure S5. Generation of GFP-tagged *P. berghei* transgenic lines in the ANKA 2.34 reference line

Schematic representation of the *PIMMS01* (*c01::gfp*) **(A)**, *PIMMS57* (*c57::gfp*) **(B)** and *PIMMS22* (*c22::gfp*) **(C)** *GFP* tagging by double crossover homologous recombination. Black arrows indicate binding sites for primers used in diagnostic PCR. **(D)** PCR genotypic analysis on mix parasite (MP) and clonal populations (CP) of the tagged transgenic parasites following transfection and dilution cloning. Used primer combinations are shown below each detected band.

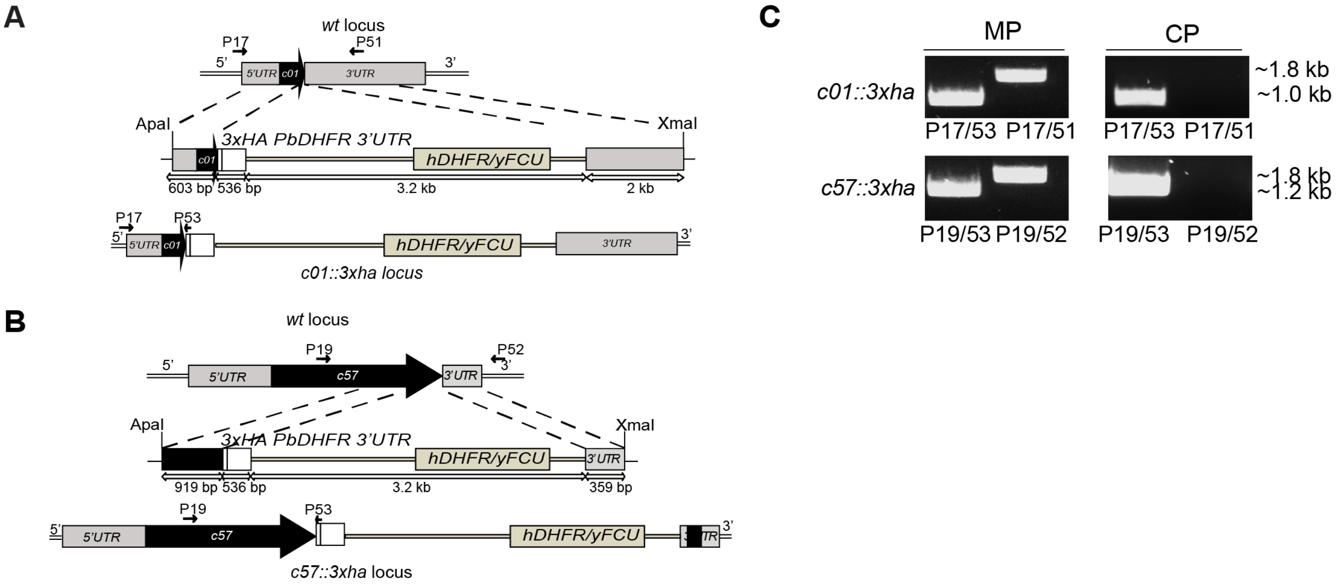

Figure S6. Generation of HA-tagged *P. berghei* transgenic lines in the ANKA 2.34 reference line

Schematic representation of the *PIMMS01* (*c01::3xha*) **(A)** and *PIMMS57* (*c57::3xha*) **(B)** *HA* tagging by double crossover homologous recombination. Black arrows indicate binding sites for primers used in diagnostic PCR. **(C)** PCR genotypic analysis on mix parasite (MP) and clonal populations (CP) of the tagged transgenic parasites following transfection and dilution cloning. Used primer combinations are shown below each detected band.

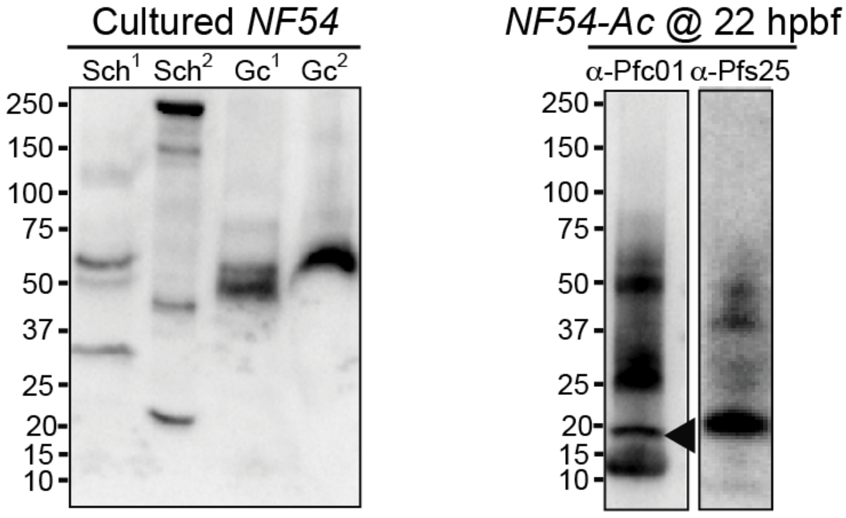

Figure S7. PfPIMMS01 expression in *in vitro* cultured schizonts and gametocytes

Western blot analysis using α-Pfc01 antibody on Triton soluble lysates ([1]), Triton insoluble cell lysates ([2]) and whole cell lysates ([3]) of *P. falciparum NF54* schizonts (Sch), gametocytes (Gc) and *A. coluzzii* midgut homogenates 22 hpbf. The PfPIMMS01 protein band is indicated with a black arrowhead. The antibody against Pfs25 (α-Pfs25) was used as a positive control in midgut homogenates.

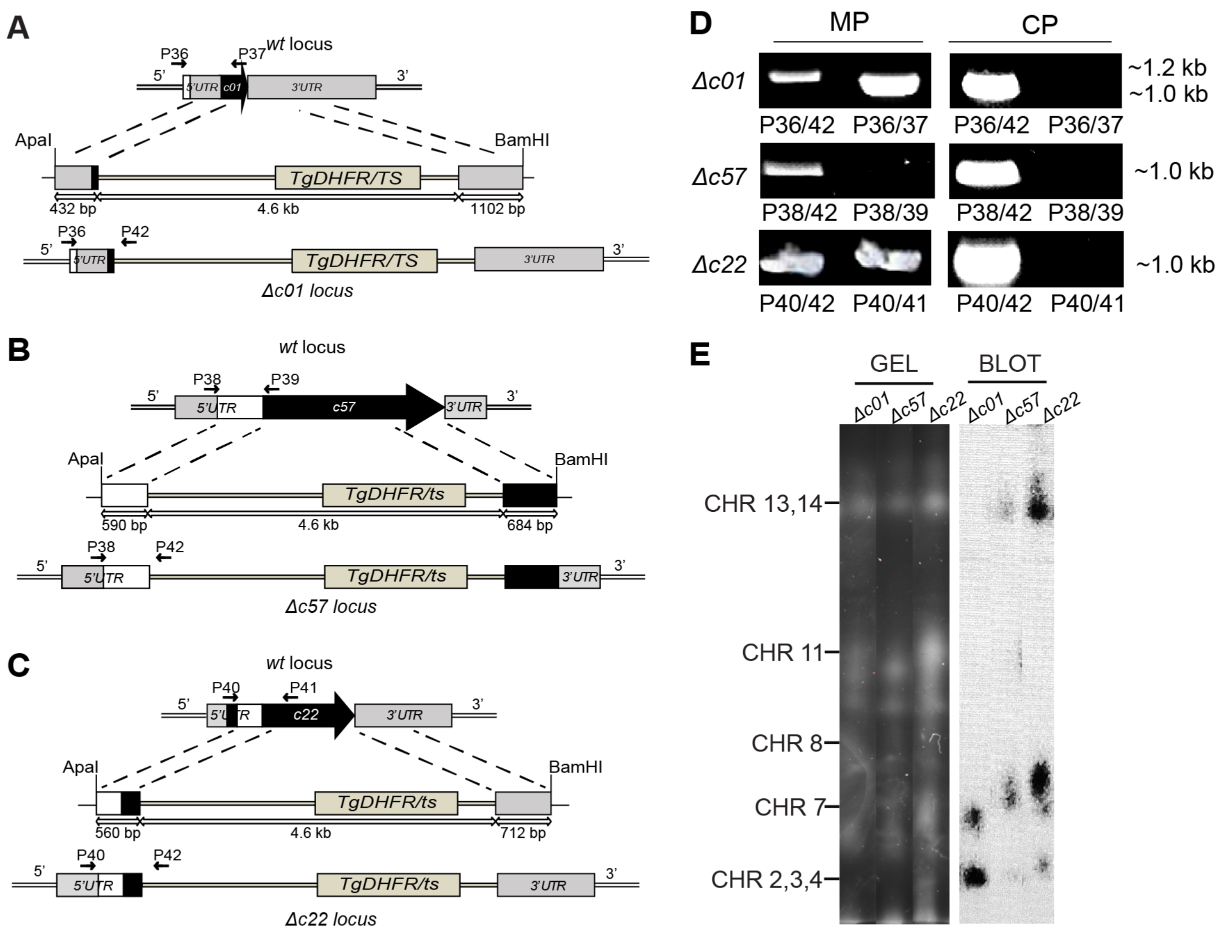

Figure S8. Generation *of P. berghei Δc01, Δc57* and *Δc22* knockout mutant lines

Schematic representation of *P. berghei PIMMS01* **(A)**, *PIMMS57* **(B)** and *PIMMS22* **(C)** locus disruption by double crossover homologous recombination in the *c507* transgenic reference line. Black arrows indicate binding sites for primers used in diagnostic PCR. **(D)** PCR genotypic analysis on mix parasite (MP) and clonal populations (CP) of the knockout mutant parasites following transfection and dilution cloning. Used primer combinations are shown below each detected band. **(E)** Southern blot analysis on pulsed field gel electrophoresis separated transgenic chromosomes.

Legends of Supplementary Movies

Supplementary Movie 1. Gliding motility of c507 ookinetes.

In vitro cultured ookinetes were embedded in Matrigel and their movement was captured with time-lapse microscopy at 20 frames per sec for 2 min.

Supplementary Movie 2. Gliding motility of *Δc01* ookinetes.

In vitro cultured ookinetes were embedded in Matrigel and their movement was captured with time-lapse microscopy at 20 frames per sec for 2 min.

Supplementary Movie 3. Gliding motility of *Δc01* ookinetes.

In vitro cultured ookinetes were embedded in Matrigel and their movement was captured with time-lapse microscopy at 20 frames per sec for 2 min.

Supplementary Movie 4. Gliding motility of *Δc57* ookinetes.

In vitro cultured ookinetes were embedded in Matrigel and their movement was captured with time-lapse microscopy at 20 frames per sec for 2 min.

Supplementary Movie 5. Gliding motility of *Δc57* ookinetes.

In vitro cultured ookinetes were embedded in Matrigel and their movement was captured with time-lapse microscopy at 20 frames per sec for 2 min.

Supplementary Movie 6. Gliding motility of *Δc57* ookinetes.

In vitro cultured ookinetes were embedded in Matrigel and their movement was captured with time-lapse microscopy at 20 frames per sec for 2 min.

Supplementary Movie 7. Gliding motility of *Δc22* ookinetes.

In vitro cultured ookinetes were embedded in Matrigel and their movement was captured with time-lapse microscopy at 20 frames per sec for 2 min.

Supplementary Movie 8. Gliding motility of *Δc22* ookinetes.

In vitro cultured ookinetes were embedded in Matrigel and their movement was captured with time-lapse microscopy at 20 frames per sec for 2 min.
