# Supplementary material for: Identification of Three Novel Plasmodium Factors Involved in Ookinete to Oocyst Developmental Transition
Source: Front Cell Infect Microbiol. 2021 Mar 15;11:634273. doi: 10.3389/fcimb.2021.634273 (PMC8005625; doi:10.3389/fcimb.2021.634273)
Supplement: Supplementary file 2 [file Table_1.pdf]

Table S1. Sporozoite numbers of *P. berghei GFP* tagged parasites in *A. coluzzii* infections.

Parasite	Mean	SEM	P-value
ANKA 2.34	5,934	2875	
c01::gfp	612	395	0.0048
c57::gfp	354	175	0.0042
c22::gfp	8,441	2422	0.4880

Mean oocyst sporozoite numbers from two biological replicates of *A. coluzzii* infections with transgenic *P. berghei* parasites carrying *GFP* tagged versions of the genes *PIMMS01*, *PIMMS57* and *PIMMS22*. For each biological replicate sporozoite numbers were determined from at least 30 homogenized mosquito midguts 15 dpbf. P values were calculated using the unpaired Student's t-test. SEM represents standard error of mean.

Table S2. Oocyst numbers of *P. berghei* HA-tagged parasites in *A. coluzzii* infections

Time	Parasite	Number of midguts	Prevalence (%)	Arithmetic mean	Median	Parasite Range	P value	Fold difference
8 dpbf	*c507*	20	75	47.9	39	0-164		
	c01::3xha	29	59	12.6	3	0-61	0.0076	3.8
	c57::3xha	35	49	3.5	0	0-22	0.0001	13.7

This table summarizes the oocyst data from two replicates of *A. coluzzii* infected with transgenic *P. berghei* parasites carrying *3xHA* tagged versions of the genes *PIMMS01* and *PIMMS57*. *P* values were calculated using the Mann-Whitney t-test. Fold differences between the transgenic parasites and the *507 wt* oocyst densities were calculated using the arithmetic mean.

Table S3. Oocyst numbers of *c507*, *Δc01*, *Δc57* and *Δc22* parasites in *A. coluzzii* infections

Time	Parasite	Number of exp	Number of midguts	Prevalence (%)	Arithmetic mean	Median	Parasite Range	P value	Fold difference
8 dpbf	*c507*	Pool	120	77	27.6	8	0-203		
	Δc01		73	52	4.3	1	0-40	<0.0001	6.4
	Δc57		89	40	1.2	0	0-14	<0.0001	23.0
	Δc22		81	56	4.5	1	0-33	<0.0001	6.1
	c507	R1	40	85	29.7	6	0-203		
	Δc01		17	65	5.9	1	0-40	0.0222	5.0
	Δc57		39	33	0.7	0	0-6	<0.0001	42.4
	Δc22		20	55	4.6	1	0-28	0.0045	6.5
	c507	R2	42	52	20.2	1	0-166		
	Δc01		26	19	1.9	0	0-20	0.0041	10.6
	Δc57		19	42	1.1	0	0-7	0.0939	18.4
	Δc22		30	30	1.2	0	0-12	0.0111	16.8
	c507	R3	38	95	33.4	24	0-137		
	Δc01		30	73	5.5	3	0-29	<0.0001	6.1
	Δc57		31	48	1.9	0	0-14	<0.0001	17.6
	Δc22		31	81	7.6	6	0-33	<0.0001	4.4

This table summarizes the oocyst data from three biological replicates of *A. coluzzii* infected with *c507*, *Δc01, Δc57* or *Δc22* parasite lines. *P* values were calculated using the Mann-Whitney t-test. Fold differences between the mutant parasites and the *507 wt* oocyst densities were calculated using the arithmetic mean.

Table S4. Sporozoite numbers of *c507*, *Δc01*, *Δc57* and *Δc22* parasites in *A. coluzzii* infections

		Midgut Sporozoites			Salivary gland Sporozoites			
Parasite	Replicates	Mean	SEM	P-value	Mean	SEM	P-value	Bite-back
c507	3	3,556	970		4,344	1,086		6/6 (2/2;2/2;2/2)
Δc01	3	256	48	0.0488	110	68	0.0337	0/6 (0/2;0/2;0/2)
Δc57	3	197	113	0.0484	36	7	0.0317	0/6 (0/2;0/2;0/2)
Δc22	3	223	24	0.0486	211	144	0.0369	0/6 (0/2;0/2;0/2)

Mean oocyst and salivary gland derived sporozoite numbers from three biological replicates of *A. coluzzii* infections. For each biological replicate (shown in brackets), sporozoite numbers was determined from 25-30 homogenized mosquito midguts or salivary glands at days 15 and 21 dpbf respectively. Infectivity of sporozoites was assessed by infected mosquito bite back experiments with at least 30 mosquitoes on C57/BL6 mice at 21 dpi. Following this, parasitaemia was monitored until 14 days post mosquito bite. *P* values were calculated using the unpaired Student's t-test. SEM represents standard error of mean.

Table S5. Invasion assay in *CTL4* knockdown *A. coluzzii*

Time	Parasite	Number of exp	Number of midguts	Prevalence (%)	Arithmetic mean	Median	Parasite range	P value
7 dpbf	*c507*	Pool	65	78	56.9	18	0-558	
	Δc01		66	70	45.2	10.5	0-419	0.3971
	Δc57		70	69	30.5	6	0-356	0.0337*
	Δc22		79	71	30.5	7	0-581	0.0487*
	c507	R1	35	83	49.7	13	0-558	
	Δc01		30	63	41.5	12.5	0-231	0.4145
	Δc57		33	70	47.6	8	0-356	0.5952
	Δc22		48	75	38.9	7	0-581	0.2605
	c507	R2	30	73	65.2	18.5	0-533	
	Δc01		36	75	48.3	10.5	0-419	0.6583
	Δc57		37	68	15.2	4	0-113	0.0299*
	Δc22		31	65	17.6	7	0-148	0.0708

Numbers of melanised parasites detected in the midguts of *c507* or *Δc01 or Δc57* or *Δc22* infected *CTL4* kd *A. coluzzii* mosquitoes at 7days post feeding. The P value was calculated using the Mann-Whitney U test. Data from pooled and independent biological replicates are presented.

Table S6. Primers for qRT-PCR and generation of transgenic parasites

Primer name	Sequence (5′ to 3′)	Description
Pbc01 qRT-PCR F	TCGCTTTATTGTTTACCATAGCATCC	
Pbc01 qRT-PCR R	TGTATGTTGCTGCTGGATTTTGTT	
Pbc57 qRT-PCR F	GCAGGCTGTTTCGTATGTTGTATT	
Pbc57 qRT-PCR R	TGTCTTTAAAAGCTTGTGCCCATT	
Pbc22 qRT-PCR F	TTCAGGATGTGAATGGGCATTTTT	
Pbc22 qRT-PCR R	TAAGAGACCCTGTGGATGACTTTG	
Pfc01 qRT-PCR F	AACCGTTTCGTTGTATTATGTCCCA	
Pfc01 qRT-PCR R	GCCTTGGCTGCATTTTCTGTT	
Pfc57 qRT-PCR F	AGAAAAGTAAATTTCGCACCACG	
Pfc57 qRT-PCR R	GTAACTGGGACCAGCATCCA	
Pfc22 qRT-PCR F	TGTTAGGTTCCAATTGTCCCAGT	
Pfc22 qRT-PCR R	AAGACCTTGTGGGTGACTTAGG	
GFP qRT-PCR F	CCTGTCCTTTTACCAGACAACCA	
GFP qRT-PCR R	GGTCTCTCTTTTCGTTGGGATCT	
Pf arginyl-tRNA synthetase qRT-PCR F	AAGAGATGCATGTTGGTCATTT	
Pf arginyl-tRNA synthetase qRT-PCR R	GAGTACCCCAATCACCTACA	
01 P1 F	TTGGGCCCGCGTTTTGTCCAATACAATAGTATGAT	GFP Tagging upstream target ApaI
01 P2 R	CCAAGCTTTAACCGTCTATTATTATTTTT	GFP Tagging upstream target HindIII
01 P3 F	TGAATTCGCACTTGCAATGAAAAAAGGAAACATAG	GFP Tagging downstream target EcoRI
01 P4 R	TTGGATCCTACTTTTTATTATTCTTATCAAT	GFP Tagging downstream target BamHI
57 P5 F	TTGGGCCCGAGTTATTAAATAGTTCTAAACAATG	GFP Tagging upstream target ApaI
57 P6 R	GGCCGCGGATAGGTTCTTTTATTGCTCATCA	GFP Tagging upstream target SacII
57 P7 F	TTCTCGAGATAATCCATGTATTACATAATGATAAT	GFP Tagging downstream target XhoI
57 P8 R	TTCCCGGGAATAAAATTTAATTTCTCTCTAAGAG	GFP Tagging downstream target XmaI
22 P9 F	TTGGGCCCGAAACAGCATTATTAATATGCATAAATG	GFP Tagging upstream target ApaI
22 P10 R	CCAAGCTTAGCATTTTCTACTGGATAAGACAGC	GFP Tagging upstream target HindIII
22 P11 F	TGAATTCGCCTTTTTATATATAAACGTTGGCG	GFP Tagging downstream target EcoRI
22 P12 R	TTGGATCCGGATCTTAAATTTGGGGTGACTATTTTC	GFP Tagging downstream target BamHI
GFP-DHFR 3'UTR (P13 F)	TTAAGCTTATGAGTAAAGGAGAAGAACTTTT	GFP tag HindIII
GFP-DHFR 3'UTR (P14 R)	TTAAGCTTCCGAAATTGAAGGAAAAAACATCATTTG	GFP tag HindIII
GFP-DHFR 3'UTR (P15 F)	GGCCGCGGATGAGTAAAGGAGAAGAACTTTT	GFP tag SacII
GFP-DHFR 3'UTR (P16 R)	GGCCGCGGCCGAAATTGAAGGAAAAAACATCATTTG	GFP tag SacII
01 tag INT F (P17)	CATGAATGCTTTAAACTTTCTACATTTAC	Diagnostic primer WT and GFP/HA tag
01 GFP tag WT R (P18)	CTAATAAACATTTCCTAATTTTTAACTTA	Diagnostic primer WT
57 tag INT F (P19)	GAGCAATAGCAATGATAAAATAATATCG	Diagnostic primer WT and GFP/HA tag
57 GFP tag WT R (P20)	GCATACATATGCATATTCATATTTCGG	Diagnostic primer WT
22 tag INT F (P21)	GTAAGTTTGTTAAATGAGAACCTTACAG	Diagnostic primer WT and GFP tag
22 tag WT R (P22)	GTTTATATATAAAAAGGCAAAGTAGCG	Diagnostic primer WT
GFP INT R (P23)	CTAATTCAACAAGAATTGGGACAACTCC	Diagnostic primer GFP tag
01 P24 F	TTGGGCCCCCAATACAATAGTATGATAC	Disruption upstream target ApaI
01 P25 R	CCAAGCTTCCTCATTATTATAACTGGATGC	Disruption upstream target HindIII
01 P26 F	TGAATTCGAATTTGAGAATCGATTATGGAGG	Disruption downstream target EcoRI

01 P27 R	TTGGATCCGATCACGCCTTTATTGAATTAAATGAAG	Disruption downstream target BamHI
57 P28 F	TTGGGCCCTAATATATTATACATAGACAATTGCTC	Disruption upstream target ApaI
57 P29 R	CCAAGCTTCATATATTGATATATGTATTTTAC	Disruption upstream target HindIII
57 P30 F	TGAATTCGGTACCATATAACTATTCTGAAGCATATG	Disruption downstream target EcoRI
57 P31 R	TTGGATCCGCTCATCATATAGAAAGTGGTATAATATATG	Disruption downstream target BamHI
22 P32 F	TTGGGCCCGATAATATATACATCCATTTTTTTG	Disruption upstream target ApaI
22 P33 R	CCAAGCTTCATATTTTCCACATAATACAGATTC	Disruption upstream target HindIII
22 P34 R	TGAATTCCTTCATGTTTGTATTTTTAAAAAGC	Disruption downstream target EcoRI
22 P35 R	TTGGATCCGGCATGTATTTAATAAAATAGCATG	Disruption downstream target BamHI
01 INT F (P36)	GGATGTATTCCTATAACTATGTG	Diagnostic primer WT and KO/*c507*
01 WT R (P37)	CTCCTTGTATGTTGCTGCTGG	Diagnostic primer WT/*c507*
57 INT F (P38)	GATGTCTAGCTAATTTGGGAATTAGTG	Diagnostic primer WT and KO/*c507*
57 WT R (P39)	GCATTATCTCTATCTTCATAATTTG	Diagnostic primer WT/*c507*
22 INT F (P40)	GAAATGGAGCATATATGTAATAAACCCG	Diagnostic primer WT and KO/*c507*
22 WT R (P41)	AAATTTGAATAAGGATTGACGCATGC	Diagnostic primer WT/*c507*
TgDHFR 5'UTR R (P42)	GATGTGTTATGTGATTAATTCATACAC	Diagnostic primer KO/*c507*
01 P43 F	GATTACGCCAAGCTTGGGCCCGCGTTTTGTCCAATACAATAGTATGATACTTAATTGTA AGCTAGCT	HA Tagging upstream target ApaI
01 P44 R	CTAGAGCGGCCGCCACCGCGGCTAAGCATAGTCTGGAACGTCATAAGGGTATGCGTA ATCTGGCACGTCGTATGGATATGCATAATCTGGTACATCGTATGGGTATAACCGTCTAT TATTATTTTTCCATGAATAA	HA Tagging upstream target SacII
01 P45 F	CTTCAATTTCGGGTACCCTCGAGAATACCTCTAAGTTAAAAATTAGGAAATGTTTATTA G	HA Tagging downstream target XhoI
01 P46 R	GAATTCGCGGCCGCCCCGGGGTTTTATAAGGTATAATGGAATATGTATGCTC	HA Tagging downstream target XmaI
57 P47 F	GATTACGCCAAGCTTGGGCCCGAGCAATAGCAATGATAAAATAATATCGGTAACAGAA GAG	HA Tagging upstream target ApaI
57 P48 R	CTAGAGCGGCCGCCACCGCGGCTAAGCATAGTCTGGAACGTCATAAGGGTATGCGTA ATCTGGCACGTCGTATGGATATGCATAATCTGGTACATCGTATGGGTAATAGGTTCTTT TATTGCTCATCATATAGAAAG	HA Tagging upstream target SacII
57 P49 F	CTTCAATTTCGGGTACCCTCGAGATAATCCATGTATTACATAATGATAAT	HA Tagging downstream target XhoI
57 P50 R	GAATTCGCGGCCGCCCCGGGAATAAAATTTAATTTCTCTCTAAGAG	HA Tagging downstream target XmaI
01 3xHA tag WT R (P51)	GTTGCGTGATAATTCATAATTACATGG	Diagnostic primer WT
57 3xHA tag WT R (P52)	GCCTTATGATGAATTAATTGTAAGTGCG	Diagnostic primer WT
3xHA INT R (P53)	GTATGCGTAATCTGGCACGTCGTATG	Diagnostic primer HA tag

Where appropriate, target restriction sites are shown as underlined. The appropriate restriction enzyme is presented in the description column. F, forward; R, reverse; INT, integration; WT, wild-type; KO, knockout; UTR, untranslated region. All primers are listed in a 5’ to 3’ direction
